# Determination and Comparison of the Lipid Profile and Sodium Content of Gluten-Free and Gluten-Containing Breads from the Spanish Market

**DOI:** 10.1007/s11130-020-00828-w

**Published:** 2020-06-02

**Authors:** Alba Tres, Natalia Tarnovska, Elisa Varona, Beatriz Quintanilla-Casas, Stefania Vichi, Anna Gibert, Elisenda Vilchez, Francesc Guardiola

**Affiliations:** 1 Nutrition, Food Science and Gastronomy Department-XaRTA, Torribera Food Science Campus, Faculty of Pharmacy and Food Science, Universitat de Barcelona, Av. Prat de la Riba, 171, 08921 Santa Coloma de Gramenet, Spain; 2 Institut de Recerca en Nutrició i Seguretat Alimentària, Universitat de Barcelona, Av. Prat de la Riba, 171, 08921 Santa Coloma de Gramenet, Spain; 3 Associació de Celíacs de Catalunya, Carrer de la Independència, 257, 08026 Barcelona, Spain

**Keywords:** Fatty acid composition, Fat, cholesterol, phytosterol and sodium content, Gluten-free bread, Nutritional value

## Abstract

The objective is to verify if gluten-free (GF) and gluten-containing (G) breads differ in their sodium content and lipid profile. Samples of GF (*n* = 20) and G (*n* = 14) sliced white sandwich bread of commercial brands most frequently consumed in Spain were collected. The fatty acid (FA) composition and the contents of sodium, fat, cholesterol and phytosterols were determined. Sodium, fat and cholesterol contents were significantly higher in GF bread. The FA composition also differed, while G breads declared in most instances the use of sunflower oil as fat ingredient and presented a higher polyunsaturated FA percentage; GF breads declared a wide variety of fats and oils as ingredients (coconut, palm, olive, sunflower, etc.) which was reflected in their FA profile. Cholesterol content was higher in GF bread because five samples declared the use of whole egg, while G samples did not include any egg product in their formulas. Phytosterol content was higher in G bread but its variability was greater in GF bread. In conclusion, nutritional quality of GF bread varied depending on the ingredients used and might be lower than that of G bread. However, these differences in composition could be reduced or eliminated through changes in the formulation of GF bread. Moreover, the comparison of the results obtained in our laboratory for fat and salt content with the declared contents on the labels showed a much higher deviation for GF samples and it can be concluded that the quality of the nutritional information declared was lower in GF samples.

**Electronic supplementary material:**

The online version of this article (10.1007/s11130-020-00828-w) contains supplementary material, which is available to authorized users.

**Abbreviations**
CD: Coeliac disease
CVD: Cardiovascular disease
DW: Dry weight
FA: Fatty acids
G: Gluten-containing
GC: Gas-chromatography
GF: Gluten-free
GFD: Gluten-free diet
ICP-OES: Inductively coupled plasma optical emission spectroscopy
MUFA: Monounsaturated fatty acids
PCA: Principal component analysis
PUFA: Polyunsaturated fatty acids
SFA: Saturated fatty acids
WW: Wet weight

## Introduction

Coeliac people represent about 1% of the European population [1]. Coeliacs must adhere to a strict gluten-free diet (GFD) for life. However, there is an increasing number of people who follow a GFD without medical prescription. In addition, not many studies have investigated the nutritional effects of the GFD on healthy populations.

Moreover, some studies carried out in some countries around the world (including Spain) using information from food labels and/or from food databases showed significant composition differences between gluten-free foods and their equivalents with gluten [2–11]. As the nutritional evaluation carried out in these studies indicated that nutritional quality of gluten-free (GF) products was lower or equivalent to that of the gluten-containing (G) products, some authors do not recommend the consumption of GF products unless there is clear evidence of gluten intolerance [9, 11]. In addition, some of these studies also recently observed that the availability of GF products on the market is still lower than that of their G counterparts [2, 4] and that their cost is higher [2, 4–6, 8]. Therefore, much research is needed to reformulate GF products and specially GF bakery products in order to improve the nutritional quality, organoleptic characteristics and consumer acceptance of the GF products present in the market [9, 12, 13].

Although several studies have found differences between the nutritional quality of GF and G products based on the labeling information, to date there are no studies in the literature that make this comparison based on the analytical results obtained from a representative market sample. Thus, to verify that these differences exist in the market, GF and G sliced white sandwich bread of commercial brands most frequently consumed in Spain were collected from local and online supermarkets and analyzed in our laboratory. The study was focused on bread because it is the most consumed GF food in Europe [14], even slightly overcoming pasta in Italy. Also, according to our previous bibliographic study, bread was the product that could present more differences in composition [10]. In addition, GF sliced white sandwich bread accounts for the 85–90% of the bread consumed by coeliacs in Spain and its gluten-containing equivalents are clearly defined and identified in the market.

Fatty acid (FA) profile and contents in fat, sodium, cholesterol and phytosterols were determined in the samples. These compositional parameters have been chosen because they are dietary factors nutritionally related with cardiovascular health. In fact, there is a controversy about if coeliac disease (CD) can predispose to cardiovascular disease (CVD) or not. On one hand, there are studies that seemed to confirm it [15, 16] but others obtained opposite results [17]. In addition, the recent systematic reviews and meta-analysis about this point [18, 19] concluded that CD could be associated to a small increase in risk for developing CVD, but the evidence base is limited and there is a need for adequately powered prospective studies with appropriate adjustment for potentially confounding factors. Another controverted aspect is the effect of the GFD on the risk of CVD in coeliacs. Some studies indicate that GFD decrease the risk [20]; while others [21] conclude that GFD does not affect this risk. A systematic review on this aspect [22] concludes that GFD alters certain cardiovascular risk factors in patients with CD, but the overall effect on cardiovascular risk is unclear and needs further comprehensive and well controlled studies. In addition, there is an even less studied and more controverted question: the effect of the GFD on CVD risk in individuals without diagnosed CD [23, 24], but the evidence at this moment indicates that GFD should be discouraged among people without CD.

The objective of this study is to compare the nutritional value of commercial sliced white sandwich bread with and without gluten, specifically with reference to its FA profile and contents in fat, sodium, cholesterol and phytosterols. In addition, the relationship between our analytical results and the list of ingredients declared on the label was studied in order to identify how the different ingredients can contribute to the composition differences found between the samples. Finally, our analytical results have been compared with the nutrition facts declared on the labels in order to assess the quality of the nutritional information provided to the consumer.

## Materials and Methods

### Sample Preparation

Samples of G (*n* = 14) and GF (*n* = 20) bread from the commercial brands most frequently consumed in Spain were collected between June and July 2017 from local and online supermarkets. All samples selected were of white sliced sandwich bread type (without any seeds or nuts). The sampling covered a market share of 85–90% for samples with gluten and of 87% for samples without gluten (for further information see supplementary material).

Two bags of bread of each sample were purchased. For each sample, 200 g (100 g from each bag) were taken, bread slices with a similar proportion of crumb and crust were taken from different positions of each bag. For all samples, no slices were taken from the ends of the bread, since a highly variable proportion of crust between samples could have affected the results. The 200 g of each sample were homogenized two times for 20 s at 3000 rpm using a knife mill (Robot Coupe Blixer 2, Vincennes, France), then packed in 7 multilayer bags (Cryovac BB325, Sealed Air SL, Sant Boi de Llobregat, Spain) and stored at −20 °C until analysis.

### Analytical Determinations

All analytical determinations were carried out in duplicate. Determination of moisture was carried according to the 925.10 AOAC method. Determination of the sodium content was conducted by inductively coupled plasma optical emission spectroscopy (ICP-OES). Determination of fat content was carried using an adaptation of the 922.06 AOAC method. Determination of the FA composition was carried by gas-chromatography (GC) after methylation of the lipid extract. Determination of the content of cholesterol and phytosterols was carried by GC after silylation of the unsaponifiable matter. For references and a detailed description of the analytical methods see supplementary material.

### Labeling Information

The declared nutritional information of all samples was recorded, analyzed and compared with the analytical results when possible. The ingredients were also recorded and a detailed list of the ingredients of each sample was prepared (supplementary material).

### Statistical Analysis

The average of all analytical duplicates was calculated and these results were used for statistical analysis.

To explore clustering of samples, a principal component analysis (PCA) was conducted with the results of all the parameters determined. The clustering was studied according to the gluten content of the samples and also according to the fats and oils declared in the list of ingredients of the samples. The PCA was carried using the SIMCA software (v13.0, Umetrics AB, Umeå, Sweden). The data was also represented for each parameter and for each type of bread (with and without gluten) using box plot diagrams (supplementary material).

The normality of the variables (determined in our laboratory and obtained from labeling) was checked using the Shapiro-Wilk test. As in some cases the variables did not follow a normal distribution, non-parametric tests (Mann-Whitney U) were used to compare the composition of G and GF bread. In all cases, the values ​​of *p* < 0.05 were considered significant. The IBM SPSS Statistics software (v23.0, IBM, Armonk, NY) was used to carry these tests.

## Results and Discussion

In all samples, moisture, FA composition and contents of sodium, fat, cholesterol and phytosterols were determined. Results for each sample are shown in Tables S1 and S2 (supplementary material).

Moisture data were used to express the contents of sodium, fat, cholesterol and phytosterols in dry weight (DW) basis. Since the differences between the results obtained for G and GF bread showed similar statistical significance regardless of whether the results were expressed on wet weight (WW) or DW basis (Table 1), we will here comment only the results on WW because they are more interesting for the consumers. Only in one GF bread sample (GF 5) the moisture value (45.58%, Table S2) ​​slightly exceeded the maximum value allowed for special breads by the Spanish legislation in 2017 (sampling dates), which was 40% for G sliced white sandwich bread and 45% for GF sliced white sandwich bread. From July 1st 2019, there are no moisture limits for these breads in the Spanish legislation. For legislation references see supplementary material.

**Table 1:** Contents in fat, sodium, cholesterol and phytosterols in gluten-containing and gluten-free samples

		Gluten-containing (*n* = 14)		Gluten-free (*n* = 20)		
		Mean ± SD	Median	Mean ± SD	Median	P*
Wet weight (WW)	Sodium (g/100 g WW)	0.44 ± 0.08	0.41	0.54 ± 0.13	0.52	0.020
	Fat (g/100 g WW)	2.0 ± 0.52	1.7	3.6 ± 1.57	3.9	<0.001
	Cholesterol (mg/100 g WW)	ND	ND	5.8 ± 10.37	tr	0.003
	Campesterol (mg/100 g WW)	3.7 ± 0.48	3.9	2.2 ± 1.36	1.8	<0.001
	Stigmasterol (mg/100 g WW)	0.3 ± 0.52	tr	1.1 ± 1.38	1.1	0.043
	*β*-Sitosterol (mg/100 g WW)	17.1 ± 2.37	17.0	10.3 ± 6.95	8.1	<0.001
	Sitostanol (mg/100 g WW)	2.8 ± 0.29	2.8	0.9 ± 0.93	1.0	<0.001
	Total phytosterols (mg/100 g WW)	23.8 ± 3.34	23.6	14.5 ± 10.24	11.3	<0.001
Dry weight (DW)	Sodium (g/100 g DW)	0.69 ± 0.12	0.64	0.89 ± 0.18	0.87	0.003
	Fat (g/100 g DW)	3.1 ± 0.78	2.7	5.9 ± 2.66	6.5	<0.001
	Cholesterol (mg/100 g DW)	ND	ND	9.3 ± 16.88	tr	0.003
	Campesterol (mg/100 g DW)	5.7 ± 0.74	6.0	3.6 ± 2.36	2.9	<0.001
	Stigmasterol (mg/100 g DW)	0.4 ± 0.80	tr	1.8 ± 2.38	1.7	0.039
	*β*-Sitosterol (mg/100 g DW)	26.7 ± 3.42	26.5	17.1 ± 11.80	13.7	0.001
	Sitostanol (mg/100 g DW)	4.4 ± 0.43	4.4	1.5 ± 1.51	1.7	<0.001
	Total phytosterols (mg/100 g DW)	37.2 ± 4.87	36.9	24.0 ± 17.43	19.1	<0.001

^*^P-values for median comparisons between gluten-containing and gluten-free samples according to Mann-Whitney U test

*ND* not detected, *tr* traces (content between the limits of detection and quantification)

**Fig. 1:**
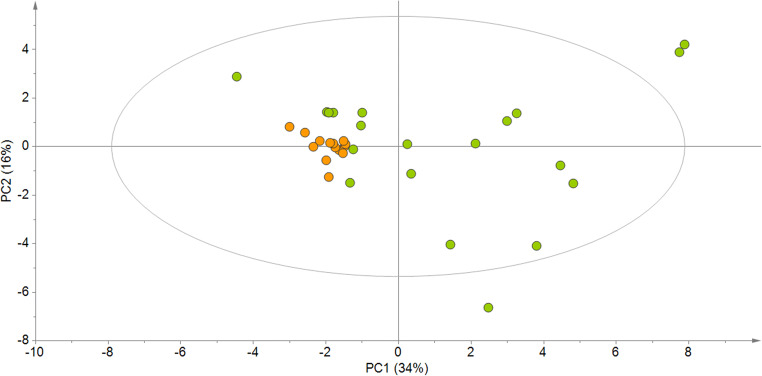
Scores plot from principal component analysis on fat, sodium, sterols and fatty acid profile data (expressed as wet weight, data mean centered and scaled to the unit of variance) from gluten-containing (*n* = 14, orange) and gluten-free (*n* = 20, green) white sandwich breads

The clustering of the samples obtained through the PCA (Fig. 1) showed a much greater variability in the composition of the GF bread samples than that of G samples.

### Sodium Content

Sodium content of GF bread was higher and more variable than that of G bread (Table 1). The values of G group ranged from 0.34 to 0.57 g Na/100 g and the values of GF samples from 0.28 to 0.75 g Na/100 g on WW. Thus, in the GF group there were both the highest (GF 7) and the lowest (GF 5) sodium content samples (Table S2).

### Fat Content

GF bread samples showed more variable fat content (from 1.5 to 7.8 g/100 g WW) than their equivalents with gluten (from 1.5 to 3.1 g/100 g WW). The median of fat content was significantly higher in GF samples (Table 1).

### Fatty Acid Composition

The FA composition of GF bread was also more variable than that of G bread, both in terms of saturated (SFA), monounsaturated (MUFA) and polyunsaturated FA (PUFA) (Table 2 and Fig. S1 in supplementary material).

**Table 2:** Fatty acid profile of gluten-containing (*n* = 14) and gluten-free (*n* = 20) white sandwich bread, results expressed as % coming from area normalization

Fatty acid (%)	Gluten-containing (*n* = 14)		Gluten-free (*n* = 20)		
	Mean ± SD	Median	Mean ± SD	Median	P*
C6:0	<0.01 ± 0.02	ND	<0.1 ± 0.02	ND	0.269
C8:0	<0.01 ± 0.01	ND	0.1 ± 025	<0.1	0.013
C10:0	ND	ND	0.1 ± 0.20	<0.01	0.015
C12:0	<0.1 ± 0.03	<0.1	0.7 ± 1.51	<0.1	0.422
C14:0	0.2 ± 0.05	0.2	0.6 ± 0.82	0.1	0.152
C15:0	<0.1 ± 0.02	<0.1	<0.1 ± 0.02	<0.1	0.802
C16:0	13.5 ± 2.16	13.8	18.3 ± 14.24	10.6	0.286
C17:0	<0.1 ± 0.03	<0.1	0.1 ± 0.04	0.1	0.920
C18:0	8.2 ± 2.71	7.6	5.0 ± 1.81	4.5	<0.001
C20:0	0.3 ± 0.05	0.3	0.3 ± 0.08	0.3	0.527
C22:0	0.6 ± 0.06	0.6	0.5 ± 0.27	0.4	0.641
C24:0	0.3 ± 0.03	0.3	0.2 ± 0.09	0.2	0.125
Total SFA	23.2 ± 4.73	23.1	25.9 ± 17.27	16.3	0.134
C16:1 *n*-7	0.2 ± 0.06	0.3	0.4 ± 0.31	0.3	0.689
C18:1 *n*-9	24.3 ± 8.63	21.3	41.4 ± 19.88	30.9	<0.001
C18:1 *n*-7	0.7 ± 0.07	0.7	1.0 ± 0.52	0.8	0.194
C20:1 *n*-9	0.3 ± 0.04	0.2	0.2 ± 0.08	0.2	0.009
Total MUFA	25.5 ± 8.66	22.5	43.1 ± 20.52	32.4	<0.001
C18:2 *n*-6	50.3 ± 8.27	53.2	30.6 ± 21.15	20.5	0.053
C20:4 *n*-6	<0.1 ± 0.03	<0.1	<0.1 ± 0.04	ND	0.071
Total *n*-6 PUFA	50.3 ± 8.27	53.2	30.6 ± 21.15	20.5	0.057
C18:3 *n*-3	1.0 ± 0.19	1.0	0.4 ± 0.31	0.2	<0.001
Total *n*-3 PUFA	1.0 ± 0.19	1.0	0.4 ± 0.31	0.2	<0.001
Ratio *n*-6/*n*-3	54.1 ± 17.32	51.0	147.0 ± 152.3	85.8	0.162
Total PUFA	51.3 ± 8.25	54.3	31.0 ± 21.05	20.7	0.028
C18:1 *trans*	<0.01 ± 0.01	ND	0.1 ± 0.09	ND	0.010

^*^P-values for median comparisons between gluten-containing and gluten-free samples according to Mann-Whitney U test

*ND* not detected, *SFA* saturated fatty acids, *MUFA* monounsaturated fatty acids, *PUFA* polyunsaturated fatty acids. The FA were quantified by peak area normalization (the quantitative results are obtained by expressing the area of a given peak as a percentage of the sum of the areas of all the identified peaks)

**Fig. 2:**
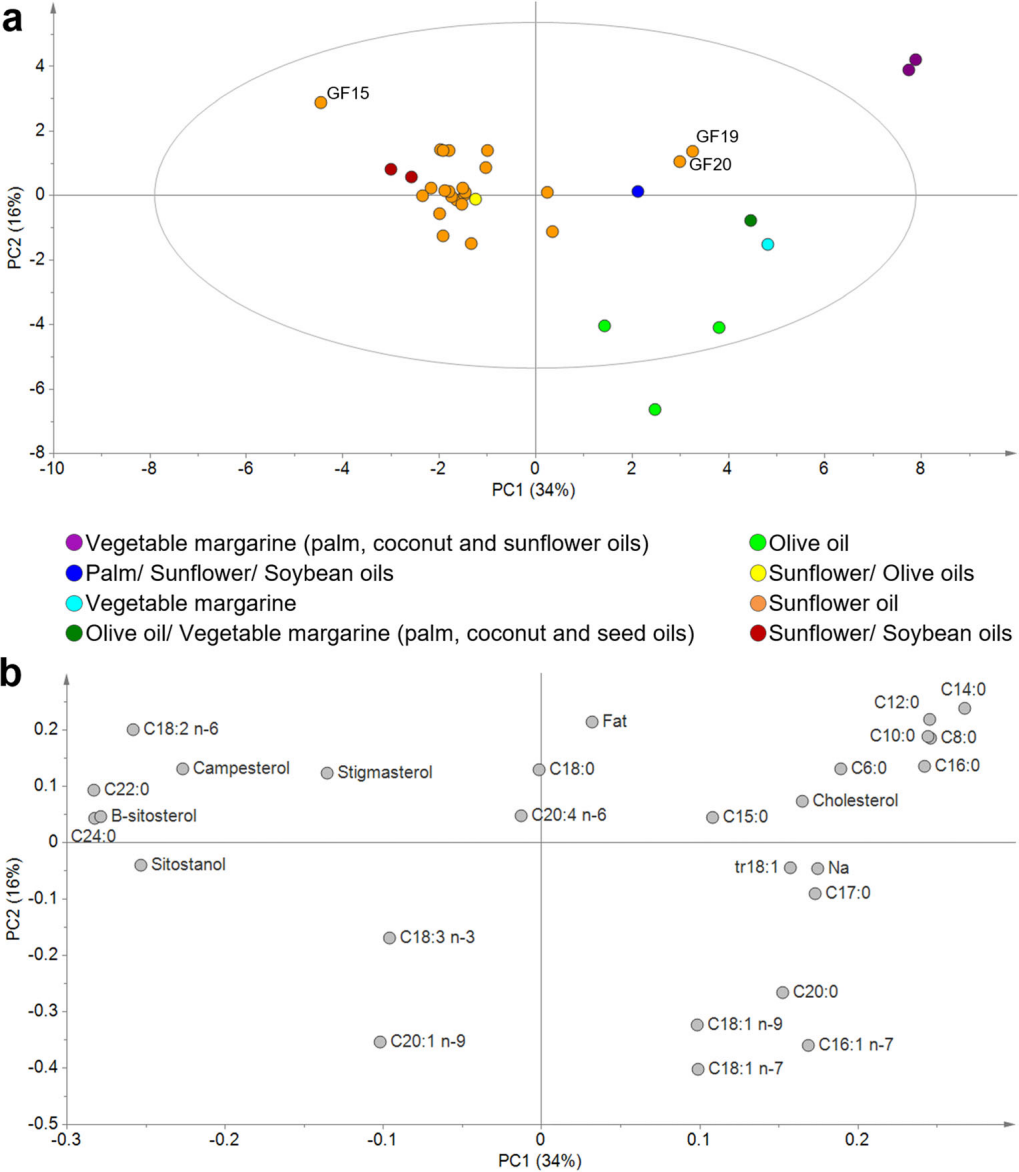
Principal components analysis on fat, sodium, sterols and fatty acid profile data (expressed as wet weight, data mean centered and scaled to the unit of variance) from gluten-containing (*n* = 14) and gluten-free (*n* = 20) white sandwich breads: (**a**) scores plot colored according to the type of fat declared in the list of ingredients; (**b**) Loadings plot of variables

The FA composition of a product such as sandwich bread depends primarily on the FA composition of the fats and oils added in its preparation, although the lipids contained in other ingredients also have a certain influence (see supplementary material for the complete list of ingredients of each sample, Tables S3 and S4). The GF samples showed a higher variability in FA composition because G samples declared only sunflower and soybean oil in their list of ingredients, while GF samples declared a variety of fats and oils (coconut, palm, olive, sunflower or soybean oils, among others) with different FA composition [25], which obviously influenced the FA composition of the final product (Fig. 2 and Table 2). In fact, in Fig. 2 it can be observed how, in general, the samples tended to be grouped according to the type of declared fat, although there were some exceptions. For instance, two samples exclusively declared sunflower oil, but appeared very separated from the rest of the samples containing sunflower oil and close to samples that used saturated fats (upper right area of Fig. 2a, GF 19 and 20).

When comparing the G and GF bread, significant differences in the total of MUFA and PUFA were found (Table 2). The G samples showed lower content in MUFA and a higher content in PUFA *n*-6 and PUFA *n*-3, in the three cases mainly due to the differences in the main FA of these groups, respectively, oleic, linoleic and linolenic acid.

The content of oleic, linoleic and linolenic acids for bread samples, in general, varied according to the fat or oil used in the formulation (see Fig. S2 for GF samples in supplementary material). For example, the GF samples that declared olive oil as an ingredient had values ​​close to 70% of oleic acid (C18:1 *n*-9) and low values ​​of linoleic acid (C18:2 *n*-6). Also, the GF sample that declared soybean oil (GF 3) among its ingredients was the one with the highest % of linolenic acid (C18:3 *n*-3) (Fig. S2c).

Regarding the GF samples that only declared sunflower oil as added fat, in general, these samples showed the highest values ​​of linoleic acid (C18:2 *n*-6) (Fig. S2b) and relatively low values of oleic (C18:1 *n*-9) (Fig. S2a). However, it should be noted that four GF samples that only declared sunflower oils as added fats did not follow this pattern. Two of these GF samples (GF 11 and 16) had abnormally high oleic acid (C18:1 *n*-9) levels and abnormally low linoleic acid (C18:2 *n*-6) levels compared to the usual FA profile of the sunflower oil [25], being their FA profile more similar to samples that declare olive oil. In fact, the FA composition of these two samples seemed to indicate that the added fat might not correspond to sunflower oil, but to an oil (or oil mixture) much richer in oleic acid and much less richer in linoleic acid, such as high-oleic sunflower oil or olive oil [25], among others. The other two GF samples that did not follow the typical FA profile of samples with only sunflower oil were GF 19 and GF 20. These samples showed lower linoleic acid values compared to the FA profile of sunflower oil and higher SFA (C12:0, C14:0, C16:0 and C18:0) values, particularly for palmitic acid (C16:0) (Figs. S2b, S4 and S5). The % of palmitic acid in these two samples ranged 46–48%, which could indicate the addition of a palmitic-rich fat such as palm oil instead of sunflower oil. These two last samples corresponded to two different brands, but came from the same producer. Thus, for some GF samples it was observed that the composition in FA was not fully explained by the type of oils declared as ingredients, which could be due to some labeling mistake.

In addition, looking at the FA composition of GF 4 that declared vegetable margarine without specifying the oils that it included, we can see that its C16:0 content was 32.8%, and thus comparable to that of the GF 1–3 samples that include palm oil in their list of ingredients (Fig. S5a). Therefore, it is very likely that this margarine contained this saturated fat among its ingredients.

On the other hand, the total SFA did not show significant differences between G and GF bread (Table 2). But, after observing the detailed profile of the various SFA, there were some significant differences in two medium-chain SFA, C8:0 and C10:0 (Table 2). Although these FA were found in low proportions, the median was higher in GF bread than in G bread. Out of 20 GF samples, GF 1, GF 2 and GF 5 presented the highest values of these FA (Fig. S3), as well as for C12:0 and C14:0 (Fig. S4). These three samples were the only ones that declared the use of coconut oil in their formulation, a fat that is richer in medium-chain SFA, especially in lauric acid (C12:0), than the other commonly used fats [25]. Due to this fact, the means for these FA in the GF group were higher, although the differences for the medians of C12:0 and C14:0 were not significant. As example, to show the magnitude of the difference, GF 1 showed 0.78% of C8:0, 0.60% of C10:0, 4.58% of C12:0 and 2.57% of C14:0, while in the rest of G and GF samples without coconut oil in their formulation the sum of these four FA was always lower than 1.5%.

The case of stearic acid (C18:0) also deserves a special comment because together with the C8:0 and C10:0, they were the three SFA that presented significant differences between the two types of bread. The values ​​of stearic acid (C18:0) were lower in GF samples (Table 2), even if samples of the two types that declared only the use of sunflower oil were compared (G samples: median = 7.7, *n* = 12; GF samples: median = 4.6, *n* = 11; *P* < 0.001). Therefore, it is possible that other ingredients than fats and oils used in bread had an influence on the values ​​of this FA. Analyzing the list of ingredients (Tables S3 and S4), it can be observed that all samples of G bread (*n* = 14) used sodium stearoyl-2-lactylate (E-481) as emulsifier, which is a source of stearic acid, whereas this additive was present only in one of the GF samples (which was not included in the previous comparison because this sample used a mixture of sunflower and extra virgin olive oils).

### Cholesterol Content

Among the samples included in the study, cholesterol was detected only in some GF samples. Thus, the content of cholesterol in GF bread was more variable and significantly higher than in G bread (Table 1 and Fig. S6).

The highest cholesterol values were between 15 and 30 mg/100 g of bread and were found in five GF samples (Table S2 and Fig. S7). These five samples were the only ones that declared the use of whole egg in the list of ingredients (see supplementary materials for the complete list of ingredients of each sample, Tables S3 and S4), and this can explain their higher cholesterol content because this ingredient is very rich in this substance [26].

Another sample (GF 15), that presented 2.2 mg cholesterol/100 g of bread, declared the use of egg white (Fig. S7). Since cholesterol is located in the egg yolk, but not in the egg white, this cholesterol content could come from small amounts of egg yolk in the used egg white.

Finally, there were traces of cholesterol in some GF samples that declared the use of egg albumin, skim milk or milk proteins in the list of ingredients (Tables S2 and S4). Cholesterol was not detected in any G sample (Table S1) because only one G sample (G 1) declared an ingredient from animal origin (whey powder) (Table S3).

Therefore, it is clear that the difference in cholesterol content existed due to the type of ingredients used in bread formulation.

Which has a certain relevancy in terms of cholesterol content, was the fact that the five GF samples that used whole egg (Fig. S7) showed a relatively high cholesterol content (between 15 and 30 mg/100 g WW) and 100 g of these breads (between 3 and 5 bread slices) were equivalent in terms of cholesterol content to 150–300 g of whole milk or to 27–54 g of pork chop or pork fillet [26]. In addition, it must be considered that among these five GF samples having the highest levels of cholesterol, three of them (GF 1, GF 2 and GF 4) are among the five GF samples having the highest SFA proportions (Table S2).

### Phytosterol Content

Phytosterols such as campesterol, stigmasterol, *β*-sitosterol and sitostanol were detected and quantified. Unlike cholesterol, all of them were found in both groups of samples. The medians of campesterol, *β*-sitosterol and sitostanol were significantly higher in G bread, while stigmasterol was higher in GF bread (Table 1). As for other parameters, the variability in the content of phytosterols was greater in GF breads (Table 1 and Fig. S8), because the ingredients used in their formulas were more numerous and variable.

Taking into account the type of fat declared in GF bread, the lowest content of phytosterols was observed in samples that contained palm and coconut oils, followed by those that contained olive oil, while it was generally the highest in samples with sunflower oil (Fig. S9). This is in agreement with the increasing content of phytosterols in sunflower or soyabean> olive> coconut or palm oils [25, 27, 28].

The richest cereals in stanols are rye, wheat, and corn [29]. Thus, the main sources of sitostanol in these bread samples were wheat and corn flours (Tables S3 and S4). The content of stanols decreases as flour refining increases. In fact, the stanols are concentrated in the outer layers of the kernel, whereas they are almost absent in the germ of these cereals [29]. This explains the higher and less variable contents of sitostanol in G samples (Table 1 and Fig. S8), which always included wheat flour (with a similar degree of refining) as ingredient, while GF samples included several ingredients derived from various GF cereals and pseudocereals (*e.g*., corn, rice, millet, teff, buckwheat, quinoa) with different refining degrees.

The difference in total phytosterols between G and GF samples was clear and significant (23.6 vs 11.3 mg/100 g WW, Table 1 and Fig. S10). This result deserves a comment because it is widely demonstrated that high contents of phytosterols in the diet reduce total- and LDL-cholesterol in plasma. However, as doses between 2 and 3 g of phytosterols and phytostanols are recommended for 2–3 weeks for a significant effect on these plasma cholesterol concentrations to be observed [30–32], the effect of the difference between G and GF samples would be minimum in relation to this effect.

On the other hand, as commented above, the content of phytosterols in the samples depended on the ingredients used (mainly the added oil), but it also depended on the fat content of the sample (the higher the fat content, usually the higher the percentage of added oil). This can be observed in Fig. S9, in which one GF bread sample (GF 15) shows much higher content of phytosterols than the rest of samples that only declare sunflower oil in their list of ingredients. However, this sample (GF 15) also showed the highest fat content by far (Table S2) among these sunflower oil-containing samples. In addition, GF 15 sample was the only one declaring rice bran as ingredient and it is well known that crude rice bran oil is one of the richest crude oils in phytosterols [33]. Thus, apart from the added oils, other ingredients, such as rice bran, could show a significant effect on the phytosterols contents. In addition, because of its high phytosterol content, this sample was separated from the rest of samples containing only sunflower oil in Fig. 2 (upper left area of Fig. 2a).

### Nutritional Information from Labeling: Comparison with the Analytical Results

The nutritional information declared on the label of each sample is shown in Tables S5 and S6 (supplementary material). First of all, to evaluate the quality of the nutritional information declared on the labels, for each sample the moisture determined in our laboratory was compared with the moisture estimated from the nutrients declared on the label of the product. The estimation was carried by the following formula %moisture = 100 – %fat – %carbohydrates – %dietary fiber – %protein – %salt. This is a rough estimation that overestimates the moisture since the ash content (that was not provided in the labels) must be higher than the declared salt content. The comparison of these moisture values showed a great difference for two GF samples (GF 18, determined, 36.8%, estimated, 14.1%; GF 20, determined 44.0%, estimated, 11.6%). Thus, the macronutrients and energy declared for these two samples would be highly overestimated and consequently were not included in Table 3 (energy was respectively the 144 and 151% of the mean energy provided by the rest of GF samples). The rest of the samples showed much lower differences between the determined and the estimated moisture. For G samples (*n* = 14) differences were: mean = −2.06%, median = −2.15%, minimum = −4.40%, maximum = 0.92%; and for GF samples (*n* = 18): mean = −0.02%; median = −0.05%; minimum = −4.53%, maximum = 6.67%.

**Table 3:** Nutritional information declared on the labels of the gluten-containing and gluten-free samples, comparison with the analytical results (100 g of edible portion)

Label information	Gluten-containing		Gluten-free		
	Mean ± SD	Median	Mean ± SD	Median	P*
Energy (kcal) (*n* = 14 *vs* 18)	254.3 ± 8.88	252.5	258.4 ± 18.01	255	0.837
Fat (g) (n = 14 vs 18)	2.9 ± 0.81	3	5.3 ± 1.94	5.1	<0.001
Saturated fat (g) (*n* = 14 *vs* 18)	0.6 ± 0.17	0.5	1.2 ± 0.85	0.9	0.001
Carbohydrates (g) (*n* = 14 *vs* 18)	46.6 ± 1.10	46	48.1 ± 7.25	48.0	0.319
Sugars (g) (*n* = 14 *vs* 18)	3.7 ± 0.89	3.8	3.3 ± 2.40	3.3	0.193
Dietary fiber (g) (*n* = 11 *vs *12)	2.8 ± 0.93	2.9	4.7 ± 2.92	4.5	0.027
Protein (g) *(n *= 14 *vs* 18)	9.2 ± 0.40	9	3.0 ± 1.65	2.4	<0.001
Salt (g) (*n* = 14 *vs* 20)	1.1 ± 0.21	1.2	1.5 ± 0.43	1.5	0.002
Analytical results and deviation relative to declared contents					
Fat (g) (*n* = 14 *vs* 20)	1.9 ± 0.53	1.7	3.6 ± 1.57	3.9	0.001
Deviation in g (*n* = 14 *vs *18)	−0.93 ± 0.51	−1.13	−1.79 ± 0.94	−1.60	0.005
Salt (g) (*n* = 14 *vs *20)	1.1 ± 0.19	1.1	1.4 ± 0.32	1.3	0.020
Deviation in g (*n* = 14 *vs* 20)	−0.001 ± 0.15	0.021	−0.107 ± 0.41	−0.009	0.500
Percentage contribution to reference nutrient intake (from analytical results)					
Fat (%) (*n* = 14 *vs* 20)	2.8 ± 0.75	2.4	5.2 ± 2.24	5.6	<0.001
Salt (%) (*n* = 14 *vs* 20)	19.1 ± 3.11	17.5	23.0 ± 5.32	22.1	0.020

^*^P-values for median comparisons between gluten-containing (G) and gluten-free (GF) samples according to Mann-Whitney U test

The number of samples used for the statistical analysis was different for each variable. For G samples *n* was always 14, except for dietary fiber that was 11, because dietary fiber content was not declared on the labels of three samples. For GF samples *n* was 20 for analytical results and declared salt content, it was 18 for the declared energy and macronutrients, except for dietary fiber that was 12 because was not declared on the labels of six samples. The energy and macronutrients declared on the label of two GF samples are not reliable, since the moisture (%) estimated from the nutrients declared greatly differs from moisture analytically determined (GF 18, determined, 36.8, estimated, 14.1; GF 20, determined 44.0, estimated, 11.6). For all samples the moisture was estimated by the following formula %moisture = 100 – %fat – %carbohydrates – %dietary fiber – %protein – %salt, and compared with the real moisture determined. Reference nutrient intakes for fat 70 g and for salt 6 g [35]

From the nutritional information declared it was observed that the differences between G and GF samples were not significant for energy, carbohydrates and sugars, and significant for the rest of nutrition facts (Table 3). Fat, saturated fat, dietary fiber and salt were higher in GF samples, while protein was lower. The previous studies analyzing labeling information of white bread reported similar significant differences for fat, dietary fiber and protein [2, 5]. However, for saturated fat Allen and Orfila [2] did not find a significant difference, while Fry et al. [5], as in our case, found higher saturated fat in GF white bread. None of these studies found a significant difference for salt content.

Our analytical results confirm that fat and salt were higher for GF samples. As both fat content and the mean SFA percentage were higher for GF samples (Tables 1 and 2), this group of samples must have on average higher saturated fat content. When the analytical results were compared with the declared nutritional information, we observed (Table 3) that determined fat was lower than the declared value and that the deviation was significantly higher for GF samples. Determined salt was lower than the declared value only for GF samples. In order to assess the deviations between determined and declared contents for each sample, the guidelines from the European Commission for the tolerances in these deviations were followed [34]. Only two G samples exceeded the tolerance in deviation for salt (Table S5); while 10 GF samples exceeded the tolerance in deviation for fat and seven for salt (Table S6). After this evaluation, it can be concluded that the quality of the nutritional information was low in 16 out of 20 GF samples; while only in two out of 14 G samples (Tables S5 and S6). The contents declared in the nutritional labeling can be obtained from analysis but they are usually calculated from the known average values of the ingredients (from ingredient specification sheets) or from generally established and accepted data in the literature [35]. Thus, the variations in the raw materials, the effect of processing, nutrient stability and storage time and conditions can affect these values. Therefore, these deviations were not surprising since the GF breads have on average much more ingredients than G breads (Tables S3 and S4) and the calculation of the declared values has more sources of uncertainty. This problem was previously anticipated by Roman et al. [13], but not checked through analytical determinations.

The fat and salt content of the G and GF samples contribute in a different proportion to the reference nutrient intakes set by the EU legislation [35] (Table 3).

## Conclusion

Samples of GF sliced white sandwich bread had higher contents of sodium, fat, cholesterol and stigmasterol, and lower contents of campesterol, *β*-sitosterol, sitostanol and total phytosterols than G samples. The FA composition was also different between sample groups, being GF bread richer in MUFA and poorer in PUFA. Regarding the SFA, significant differences were observed for some concrete FA such as C8:0 and C10:0, with higher percentage in GF bread, or in C18:0, with a higher percentage in G bread. The differences between samples in sterol and fatty acid profiles depended on the ingredients used in their formulation.

In conclusion, the nutritional quality of GF bread varied according to the ingredients used in its preparation and might be lower than that of G bread. It could be discussed whether these differences in composition are sufficiently important from a nutritional point of view when GF bread is included in an adequate and balanced diet, but this discussion does not make much sense, because these differences in composition could be easily reduced or eliminated by changes in the formulation of GF bread. For instance, since 13 of the 20 GF samples did not contain cholesterol or only contained traces of this component, it is clear that GF bread without cholesterol can be formulated. In fact, except in five GF samples where whole egg was used, cholesterol levels were practically negligible. Also, the reduction of sodium in GF samples can be feasible because 9 of the 20 GF samples had sodium content below 0.5 g/100 g WW. On the other hand, only four of the 20 GF samples declared the use of saturated fats (Table S4); however, the FA composition suggested that seven of the 20 GF samples included fats such as coconut and palm oils (Figs. S3, S4 and S5). In addition, if we look at the composition of the GF samples 10 and 11, we see that their composition is very close to the average composition values of G samples, therefore the reformulation of the GF bread is completely feasible. Of course, the reformulation is a task that can be more or less complicated based on the knowledge of the technological and nutritional functions of the ingredients and on empiric observations and experiments (usually, trial/error experiments until achieving good organoleptic characteristics, consumer acceptance and nutritional quality). In addition, it is also possible to educate coeliacs to use the ingredient list and nutrition information on the label to choose the highest quality breads, which also will push the industry to reformulation of GF products.

In addition, the comparison of the analytical results obtained in our laboratory for fat and salt content with the declared contents on the labels showed a much higher deviation for GF samples and it can be concluded that the quality of the nutritional information declared was lower in GF samples.

## Electronic supplementary material

ESM 1 (PDF 1003 kb)
