# Supplementary material for: Determination and Comparison of the Lipid Profile and Sodium Content of Gluten-Free and Gluten-Containing Breads from the Spanish Market
Source: Plant Foods Hum Nutr. 2020 Jun 2;75(3):344–54. doi: 10.1007/s11130-020-00828-w (PMC7378101; doi:10.1007/s11130-020-00828-w)
Supplement: Supplementary file 1 — (PDF 1003 kb) [file 11130_2020_828_MOESM1_ESM.pdf]

Supplementary material

Determination and comparison of the lipid profile and sodium content of gluten-free and gluten-containing breads from the Spanish market

Alba Tres[1,2], Natalia Tarnovska[1], Elisa Varona[1,2], Beatriz Quintanilla-Casas[1,2], Stefania Vichi[1,2], Anna Gibert[3], Elisenda Vilchez[3], Francesc Guardiola*[1,2]

[1] Nutrition, Food Science and Gastronomy Department-XaRTA, Torribera Food Science Campus, Faculty of Pharmacy and Food Science, Universitat de Barcelona, Av. Prat de la Riba, 171. 08921 Santa Coloma de Gramenet, Spain

[2] Institut de Recerca en Nutrició i Seguretat Alimentària, Universitat de Barcelona, Av. Prat de la Riba, 171. 08921 Santa Coloma de Gramenet, Spain

[3] Associació de Celíacs de Catalunya, Carrer de la Independència, 257, 08026 Barcelona, Spain

*Corresponding author: fguardiola@ub.edu; Tel.: 34-93-4037196. ORCID: 0000-0002-8624-8749.

Sampling representativeness

According to the last data available from 2012 and 2013, the market share in Spain for sliced sandwich bread (calculated from the tons sold) was respectively 26.2 and 24.8 % for Bimbo Co., 9.2 and 7.9 % for Panrico Co., 62.2 and 64.8% for the supermarket own brands and 2.4 and 2.5% for the rest of the companies (https://elpais.com/economia/2014/10/31/actualidad/1414781793_856001.html). Therefore, gluten-containing samples of sliced white sandwich bread from the 3 trademarks (Bimbo®, Panrico® and Ortiz®) with the highest market share and from 11 own brand breads corresponding to the supermarket chains with the highest market share in Spain were taken. The market share of the supermarket chains was calculated based on their turnover in 2016 (https://www.distribucionactualidad.com/wp-content/uploads/2017/07/daretail_supers_2017.pdf). Subsequently, data were published regarding 2017, largely coinciding with those of 2016 (http://agricultura.gencat.cat/web/.content/de_departament/de02_estadistiques_observatoris/27_butlletins/02_butlletins_nd/documents_nd/fitxers_estatics_nd/2018/0217_2018_IA_Distribucio_Espanya-CESCE-2017.pdf). In total, it is estimated that the market share covered by the sampling for gluten-containing sliced sandwich bread was between 85-90%, depending on the data used for its calculation.

Regarding the sampling of the gluten-free sliced sandwich bread, it was designed by the Coeliac Association of Catalonia and is estimated to cover the 87% of the market share. The design was based on the deep knowledge of the Spanish market that the association has through the biannual preparation for their associates of the list of gluten-free foods available.

Analytical determinations

All analytical determinations were carried out in duplicate.

- *Determination of moisture* was carried according to the 925.10 AOAC method [1] in an air recirculation oven at 130 °C.
- *Determination of the sodium content* was conducted by inductively coupled plasma optical emission spectroscopy (ICP-OES) in a Perkin Elmer Optima 8300. The calibration was done with

five standards prepared from certified standard solutions traceable to NIST. The samples (0.1 g) were previously digested with HNO_3 and H_2O_2 in a Teflon reactor heated in an oven at 90 °C.

- *Determination of fat content* was carried using an adaptation the 922.06 AOAC method [1]. The adaptation consisted in using 50 mL screw-capped centrifuge tubes for acid hydrolysis and fat extraction of samples. Extraction was repeated 5 times with petroleum ether (20 mL x 2 and 15 mL x 3). The results obtained by using this method were compared with the results obtained by using the Soxtec method with petroleum ether (AOAC 2003.06) [1] without acid hydrolysis. It was observed that the method that extracted the fat after the acid hydrolysis gave higher percentages of fat, particularly in breads with low fat content, and therefore we decided to use this method to determine fat content in all the samples.
- *Determination of the composition in fatty acids* (FA) was carried by gas-chromatography after the extraction of the lipid fraction from 2 grams of bread with hexane: isopropanol (3:2, v/v) three times (20 mL x 1 and 10 mL x 2) using a high-speed homogenizer. Determination was conducted according to a modification of the method described by Guardiola et al. [2]. This method uses double methylation in methanolic medium, first with sodium methoxide and then with boron-trifluoride. This 2-step procedure (base and acid catalyzed methylation) allows the complete methylation of free FA. The fatty acid methyl esters (FAME) were analyzed on an Agilent 4890D model (Waldbronn, Germany) gas chromatograph equipped with a flame-ionization detector (FID) and a split-splitless injector, set at 300 and 270 °C, respectively. The split ratio was 1:30. Chromatographic separation of FAME was performed on a fused-silica capillary column (60 m × 0.25 mm i.d.) coated with 0.2 μm of a stationary phase of 90% biscyanopropyl-/10% cyanopropylphenyl-polysiloxane (SP-2380 from Supelco Inc., Bellefonte, PA, USA). Hydrogen, at 25 psi, was used as a carrier gas. The program of the oven was: firstly 1 min at 150 °C, then increased the temperature by 3 °C/min to 180 °C at which point it is held for 0.5 min; then by 14.5 °C/min to 220 °C at which point it is held for 3 min, and then finally the temperature was increased by 9.9 °C/min to 250 °C and held for 9 min and 30 seconds at this temperature. The sample volume injected was 1 μL. The FA were quantified by peak area normalization (the quantitative results are obtained by expressing the area of a given peak as a percentage of the sum of the areas of all the identified peaks).

- *Determination of the content of cholesterol and phytosterols*, 1 gram of bread was weighted in a 50 mL screw-capped centrifuge tube, 100 uL of 5α-cholestane (1 mg/mL) as internal standard and 5 mL of ethanolic antioxidant solution (0.012% BHT, 0.4% anhydrous citric acid, 1% pyrogallol) were added. Then, the content was homogenized with a Polytron PT 3100 (Kinematica, Lucerne, Switzerland) at 20000 rpm during 30 seconds. Afterwards, 10 mL of potassium hydroxide in methanol (9%, w/v) was added and the mixture was vortexed and placed in a water bath at 70 °C for 30 min under gentle agitation. Then, tubes were placed for 5 minutes in an ice bath, and 15 mL of deionized water was added. After that, the unsaponifiable was extracted three times with 10 mL of cyclohexane. Once the solvent was evaporated, the unsaponifiable residue was re-dissolved with 2 mL of pyridine and an aliquot part of 50 μL was derivatized with 50 μL Sylon BTZ (Supelco, Inc.) for sterol determination. Determination was achieved by GC-FID on an Agilent 5890 model (Waldbronn, Germany) gas chromatograph. Chromatographic separation was conducted on a Zerbon™ ZB-5MS (5% polysilarylene/95% dimethyl-polysiloxane phase) capillary column (60 m x 0.25 mm i.d., 0.25 μm) from Phenomenex (Torrance, CA, USA). Hydrogen at 20 psi was used as carrier gas. One microliter of sample was injected in a split-splitless injector set at 290 °C (1:50 split ratio). Detector was set at 350 °C. The oven was set at 245 °C for 0.5 min; then temperature was increased at 2.5 °C/min to 264 °C; then at 4.2 °C/min to 290 °C at which temperature was held for 36 min. The quantification was carried out using 5α-cholestane as internal standard and the corresponding calibration curves with standards. The limits of detection and quantification for cholesterol were of 0.15 mg/100 g and 0.50 mg/100 g bread, respectively.

Table S1 Contents of moisture, sodium, fat, cholesterol and phytosterols, and the fatty acid composition of gluten-containing (G) sliced white sandwich bread samples

G samples	G1	G2	G3	G4	G5	G6	G7	G8	G9	G10	G11	G12	G13	G14
Moisture (%)	35.4	33.5	35.5	36.4	36.7	36.3	36.2	36.8	36.8	36.3	36.3	36.3	35.3	35.3
Na (g/100 g WW)	0.51	0.41	0.55	0.40	0.49	0.35	0.57	0.41	0.39	0.57	0.41	0.39	0.43	0.42
Fat (g/100 g WW)	3.14	3.01	1.72	1.54	1.97	2.07	1.76	1.68	1.52	1.63	1.61	1.70	2.30	1.57
Cholesterol (mg/100g WW)	ND	ND	ND	ND	ND	ND	ND	ND	ND	ND	ND	ND	ND	ND
Campesterol (mg/100g WW)	4.17	4.06	3.81	3.92	2.74	3.47	3.91	4.29	3.07	3.51	3.91	4.23	3.42	3.09
Stigmasterol (mg/100g WW)	1.38	1.32	tr	0.90	tr	tr	tr	tr	tr	tr	tr	tr	tr	tr
β-sitosterol (mg/100g WW)	21.55	22.10	17.27	16.12	14.41	16.37	16.98	18.20	13.65	16.30	17.47	17.05	17.15	14.78
Sitostanol (mg/100g WW)	3.03	3.12	2.90	2.65	2.54	2.79	2.51	3.26	2.45	2.58	2.85	3.03	3.02	2.31
**FA Composition (%)**														
C4:0	ND	ND	ND	ND	ND	ND	ND	ND	ND	ND	ND	ND	ND	ND
C6:0	ND	ND	ND	ND	ND	ND	ND	ND	ND	ND	ND	ND	0.07	ND
C8:0	ND	ND	ND	ND	ND	ND	ND	ND	ND	ND	ND	ND	0.03	ND
C10:0	ND	ND	ND	ND	ND	ND	ND	ND	ND	ND	ND	ND	ND	ND
C12:0	0.02	0.03	0.10	0.05	0.04	0.05	0.08	0.05	0.04	0.05	0.10	ND	0.03	0.05

C14:0	0.12	0.11	0.25	0.18	0.27	0.17	0.27	0.16	0.12	0.22	0.19	0.21	0.19	0.17
C15:0	0.02	0.03	0.03	0.06	0.06	0.04	ND	0.02	ND	0.05	0.08	ND	0.04	0.04
C16:0	10.17	9.63	15.52	15.07	13.77	13.56	14.09	13.93	11.41	13.89	12.88	18.21	13.74	12.97
C17:0	0.04	0.05	0.04	0.08	0.08	0.07	0.07	0.08	0.06	0.07	0.16	0.14	0.07	0.06
C18:0	4.89	5.13	9.08	10.46	6.84	8.30	7.50	10.15	7.41	7.78	6.75	15.87	7.61	7.03
C20:0	0.28	0.27	0.41	0.44	0.29	0.28	0.28	0.32	0.29	0.32	0.32	0.34	0.34	0.30
C22:0	0.65	0.71	0.59	0.62	0.60	0.59	0.55	0.57	0.70	0.58	0.65	0.49	0.66	0.59
C24:0	0.26	0.29	0.24	0.31	0.27	0.25	0.23	0.29	0.31	0.24	0.31	0.21	0.27	0.27
**Total SFA**	16.45	16.27	26.26	27.27	22.22	23.32	23.09	25.58	20.33	23.21	21.44	35.46	23.05	21.49
C16:1 n-7	0.25	0.19	0.28	0.33	0.19	0.21	0.36	0.25	0.25	0.27	0.19	0.27	0.14	0.19
C18:1 n-9	27.33	24.98	20.27	20.32	21.77	21.04	21.34	21.73	52.93	20.51	20.12	19.20	26.76	21.23
C18:1 n-7	0.78	0.73	0.70	0.70	0.80	0.70	0.76	0.71	0.75	0.70	0.71	0.59	0.92	0.72
C20:1 n-9	0.22	0.23	0.23	0.32	0.27	0.27	0.23	0.27	0.33	0.23	0.23	0.25	0.36	0.25
**Total MUFA**	28.61	26.13	21.48	21.66	23.03	22.23	22.70	22.96	54.27	21.71	21.25	20.30	28.18	22.39
C18:2 n-6	54.27	56.95	51.15	49.73	53.91	53.35	53.01	50.46	24.47	54.11	56.35	43.34	47.36	55.06
C20:4 n-6	0.65	0.62	1.08	1.23	0.84	1.07	1.13	0.98	0.93	0.93	0.97	0.90	1.31	1.02
C18:3 n-3	0.02	0.03	0.02	0.10	ND	0.04	0.08	0.02	ND	0.03	ND	ND	0.09	0.05
**Total PUFA**	54.94	57.60	52.25	51.07	54.75	54.45	54.22	51.46	25.40	55.08	57.31	44.24	48.76	56.12
C18:1 *trans*	0.03	ND	ND	ND	ND	ND	ND	ND	ND	ND	ND	ND	ND	ND

ND, not detected (value under the detection limit); tr, traces (value between detection and quantification limit). WW, wet weight. SFA, saturated fatty acids. MUFA, monounsaturated fatty acids. PUFA, polyunsaturated fatty acids

Table S2 Contents of moisture, sodium, fat, cholesterol and phytosterols, and the fatty acid composition of gluten-free (GF) sliced white sandwich bread samples

GF samples	GF1	GF2	GF3	GF4	GF5	GF6	GF7	GF8	GF9	GF10	GF11	GF12	GF13	GF14	GF15	GF16	GF17	GF18	GF19	GF20
Moisture (%)	34.9	32.5	34,3	35.7	45.6	39.8	39.2	35.0	40.7	40.0	36.7	35.8	42.5	42.6	42.1	40.0	42.4	36.8	41.9	44.0
Na (g/100 g WW)	0.70	0.67	0.53	0.72	0.28	0.62	0.75	0.63	0.53	0.36	0.47	0.49	0.48	0.46	0.48	0.43	0.47	0.71	0.56	0.51
Fat (g/100 g WW)	4.36	4.77	1.73	1.76	1.99	1.46	2.59	4.91	5.14	1.84	2.69	4.06	2.59	3.95	7.78	3.04	3.93	4.52	4.73	4.26
Cholesterol (mg/100g WW)	17.72	17.33	0.78	28.93	ND	ND	tr	ND	tr	ND	tr	ND	29.82	tr	2.22	19.31	tr	ND	ND	ND
Campesterol (mg/100g WW)	1.87	1.99	1.61	1.27	1.34	1.01	1.42	3.08	2.66	1.26	1.96	2.26	1.66	2.68	7.30	1.41	2.64	2.99	1.24	1.55
Stigmasterol (mg/100g WW)	tr	tr	1.23	tr	tr	tr	tr	1.49	1.70	1.22	1.53	1.45	0.78	2.17	5.96	0.91	1.89	1.69	tr	tr
β-sitosterol (mg/100g WW)	3.40	3.72	5.96	2.02	5.57	7.30	8.52	16.57	16.10	9.05	14.59	11.66	7.67	14.01	31.75	6.43	13.79	17.58	5.03	5.57
Sitostanol (mg/100g WW)	tr	tr	tr	tr	tr	1.39	1.21	1.80	1.19	1.12	1.46	0.92	1.34	0.98	2.48	tr	0.92	3.26	tr	tr
**FA Composition (%)**																				
C4:0	ND	ND	ND	ND	ND	ND	ND	ND	ND	ND	ND	ND	ND	ND	ND	ND	ND	ND	ND	ND
C6:0	0.06	0.06	0.04	ND	0.05	ND	0,03	ND	ND	ND	ND	0,03	ND	ND	ND	ND	ND	0,02	ND	ND
C8:0	0.78	0.76	0.07	0.05	0.47	ND	0.25	ND	ND	0.34	ND	0.04	ND	ND	ND	ND	0.03	0.02	0.02	0.03
C10:0	0.60	0.65	0.06	ND	0.36	ND	0.20	ND	ND	0.24	ND	0.02	ND	ND	ND	ND	0.02	ND	0.02	0.01
C12:0	4.58	4.83	0.42	0.24	2.74	ND	0.04	ND	ND	0.02	0.04	0.03	ND	ND	ND	0.03	0.03	0.02	0.24	0.22

C14:0	2.57	2.70	0.87	0.77	1.42	0.05	ND	ND	0.09	0.13	0.07	0.12	0.14	0.09	0.11	0.09	0.10	ND	0.91	0.86
C15:0	0.05	0.05	0.04	0.07	0.03	0.04	0.03	ND	ND	0.02	0.03	0.04	0.04	0.03	ND	0.08	0.03	0.03	0.06	0.06
C16:0	36.64	36.49	28.17	32.78	21.27	13.31	13.62	11.22	8.97	7.47	4.90	8.15	10.03	7.28	7.68	6.78	6.93	9.90	47.98	46.06
C17:0	0.10	0.10	0.10	0.15	0.08	0.11	0.08	0.10	ND	0.02	0.04	0.06	0.09	0.06	ND	0.07	0.05	ND	0.12	0.12
C18:0	9.08	8.80	4.22	5.29	3.92	3.66	4.55	4.10	4.26	3.34	2.68	5.48	4.83	4.57	3.62	3.49	4.54	5.03	8.09	6.94
C20:0	0.36	0.34	0.34	0.31	0.42	0.51	0.44	0.47	0.32	0.28	0.30	0.30	0.25	0.27	0.27	0.25	0.27	0.29	0.38	0.39
C22:0	0.15	0.15	0.29	0.18	0.21	0.27	0.13	0.22	0.57	0.70	0.82	0.76	0.61	0.78	0.68	0.81	0.78	0.73	0.30	0.31
C24:0	0.10	0.10	0.15	0.14	0.12	0.17	0.07	0.15	0.22	0.27	0.36	0.29	0.24	0.29	0.28	0.31	0.28	0.29	0.12	0.12
**Total SFA**	55.07	55.03	34.76	39.97	31.10	18.12	19.44	16.24	14.44	12.86	9.24	15.33	16.23	13.37	12.65	11.91	13.07	16.34	58.24	55.11
C16:1 n-7	0.36	0.36	0.23	0.74	0.51	1.19	1.00	0.70	0.35	0.14	0.16	0.16	0.57	0.13	0.14	0.51	0.12	0.16	0.22	0.25
C18:1 n-9	30.26	30.09	32.48	38.22	55.44	67.88	69.81	70.51	39.63	29.04	78.56	29.35	31.62	26.76	24.26	75.60	27.33	27.97	20.96	22.43
C18:1 n-7	0.66	0.65	0.81	1.08	1.42	2.35	2.10	1.85	1.16	0.77	0.85	0.71	0.97	0.70	0.72	0.92	0.70	0.75	0.50	0.54
C20:1 n-9	0.13	0.12	0.16	0.21	0.25	0.42	0.23	0.32	0.20	0.24	0.33	0.16	0.16	0.16	0.18	0.27	0.17	0.17	0.10	0.10
**Total MUFA**	31.48	31.34	33.68	40.63	57.63	71.84	73.14	73.58	41.33	30.18	80.01	30.38	33.32	27.76	25.30	77.30	28.36	29.10	21.85	23.41
C18:2 n-6	13.27	13.40	30.24	18.96	10.55	9.23	6.76	9.61	43.84	56.29	10.46	54.15	50.21	58.74	61.89	10.47	58.43	54.39	19.73	21.24
C20:4 n-6	0.16	0.16	1.31	0.44	0.71	0.81	0.64	0.57	0.39	0.56	0.29	0.13	0.25	0.14	0.16	0.20	0.15	0.16	0.19	0.24
C18:3 n-3	0.02	0.07	0.01	ND	0.02	ND	0.01	ND	ND	0.10	ND	0.01	ND	ND	ND	0.12	ND	0.01	ND	ND
**Total PUFA**	13.45	13.63	31.56	19.40	11.28	10.04	7.42	10.18	44.23	56.96	10.75	54.29	50.45	58.88	62.05	10.79	58.58	54.57	19.91	21.48
C18:1 *trans*	0.08	0.11	ND	0.38	ND	ND	ND	0.20	ND	ND	0.13	ND	ND	ND	ND	ND	0.04	0.05	0.08	0.08

In yellow the value of humidity that slightly exceeds the limit established until July 2019 for gluten-free bread (45%) [3, 4]. For abbreviations see Table S1.

Table S3 List of ingredients as declared in the labels of the different gluten-containing (G) sliced white sandwich bread samples

G Samples	List of ingredients
**G1**	Wheat flour, water, **vegetable oils (sunflower, soybean) in variable proportion**, yeast, sugar, salt, oat fiber, emulsifiers (E-481, E-471), preservatives (E-200, E-282), soy flour , whey powder, wheat gluten, thickener (guar gum), flour treatment agent (ascorbic acid), flavour.
**G2**	Wheat flour, water, sugar, **vegetable oils (soybean, sunflower) in variable proportion**, yeast, salt, bean flour, emulsifiers (E-481, E-471), preservatives (E-200, E-282), wheat gluten, thickener (guar gum), flour treatment agent (ascorbic acid), flavour.
**G3**	Wheat flour (58%), water, yeast, **sunflower oil**, salt, sugar, emulsifiers (E-472e, E-471, E-481), preservatives (E-282, E-200), acidity regulator (lactic acid), flour treatment agents (ascorbic acid, L-cysteine).
**G4**	Wheat flour, water, yeast, sugar, salt, **sunflower oil**, emulsifiers (E-481, E-471, E-472), wheat gluten, preservatives (E-282, E-200), wine vinegar, bean flour, acidity regulator (calcium phosphates), flour treatment agent (ascorbic acid).
**G5**	Wheat flour (58%), water, yeast, **sunflower oil (1%)**, salt, sugar, emulsifiers (E-472e, E-471, E-481), preservatives (E-282, E-200), acidity regulator (lactic acid), flour treatment agent (ascorbic acid).
**G6**	Wheat flour, water, yeast, **sunflower oil (1%)**, sugar, oat fiber, salt, emulsifiers (E-481, E-471), thickener (guar gum), preservatives (E-200, E-282), wheat gluten, soy flour, flour treatment agent (ascorbic acid), flavour.
**G7**	Wheat flour, water, yeast, sugar, **sunflower oil (1.8%)**, salt, emulsifiers (E-472e, E-471, E-481), preservatives (E-281, E-202).
**G8**	Wheat flour (57%), water, sugar, yeast, **sunflower oil (1%)**, emulsifiers (E-472e, E-471, E-481), salt, preservatives (E-282, E-200), bean flour, acidity regulator (lactic acid), flour treatment agents (ascorbic acid, L-cysteine).
**G9**	Wheat flour, water, yeast, sugar, **sunflower oil (1%)**, salt, wine vinegar, bean flour, emulsifiers (E-472e, E-471, E-481), flour treatment agent (ascorbic acid), preservatives (E-282, E-202, E-200).
**G10**	Wheat flour, water, yeast, sugar, **sunflower oil**, salt, emulsifiers (E-472e, E-471, E-481), preservatives (E-282, E-200), acidity regulator (lactic acid), flour treatment agent (ascorbic acid).
**G11**	Wheat flour, water, yeast, **sunflower oil (1%)**, salt, sugar, emulsifiers (E-472e, E-471, E-481), preservatives (E-282, E-200), acidity regulator (lactic acid), flour treatment agent (ascorbic acid).
**G12**	Wheat flour, water, yeast, sugar, **sunflower oil**, salt, wheat gluten, emulsifiers (E-471, E-481, E-472), preservatives (E-282, E-200), wine vinegar, bean flour, acidity regulator (calcium phosphates), flour treatment agent (ascorbic acid).
**G13**	Wheat flour (69%), water, yeast, sugar, **sunflower oil (1.6%)**, bean flour, salt, emulsifiers (E-471, E-481), preservatives (E-200, E-282), vinegar, thickener (guar gum), flour treatment agent (ascorbic acid).
**G14**	Wheat flour (68%), water, yeast, sugar, **sunflower oil**, salt, bean flour, vinegar, preservatives (E-282, E-200), emulsifiers (E-471, E-481), thickener (guar gum), flour treatment agent (ascorbic acid).

In bold letters the oils added to each bread sample

Table S4 List of ingredients as declared in the labels of the different gluten-free (GF) sliced sandwich bread samples

GF Samples	List of ingredients
**GF1**	Corn starch (42%), water, sugar, **liquid pausterised egg, vegetable margarine [tropical oils (palm, coconut), sunflower oil, water, emulsifier (E-471), acidity regulator (citric acid), preservative (E-202), flavours, colour (beta-carotene)]**, yeast, thickener (xanthan gum), emulsifier (E-472e), salt, raising agents (E-450i, E-500ii), preservative (E-281), ascorbic acid.
**GF2**	Corn starch, water, sugar, **liquid pausterised egg, vegetable margarine [tropical oils (palm, coconut), water, sunflower oil, emulsifier (E-471), acidity regulator (citric acid), preservative (E-202), flavours, colour (carotenes)]**, yeast, thickener (xanthan gum), emulsifier (E-472e), salt, preservative (E-281), ascorbic acid, raising agents (E-450i, E-500ii).
**GF3**	Wheat starch (59%), corn starch, milk proteins, soy flour, sugar, yeast, **palm oil, seed oils (sunflower, soybean)**, salt, egg albumin, anti-caking agent (calcium carbonate), humectant (sorbitol), preservative (E-202), ascorbic acid.
**GF4**	Gluten-free blend [corn starch, **egg**, sugar, thickener (xanthan gum), salt, acidity regulator (calcium phosphates), ascorbic acid], water, **vegetable margarine**, sugar, **egg**, yeast, salt.
**GF5**	Water, corn starch, sea water, rice flour, yeast, tapioca starch, modified starch, **olive oil**, honey, **vegetable margarine with a 80% of oil [tropical oils (palm, coconut in different proportions), seed oils (soybean, sunflower, rapeseed and corn in different proportions), water, emulsifiers (E-471, E-475, soy lectin), preservative (E-202), acidity regulator (citric acid), flavour, colour (beta-carotene)]**, thickeners (cellulose, xanthan gum), ascorbic acid
**GF6**	Gluten-free flour blend [corn starch, buckwheat flour (7%), teff flour (1%), quinoa flour, chickpea flour], water, **virgin olive oil**, thickener (xanthan gum), agave syrup, rice syrup, vegetable fiber (2%), salt, apple cider vinegar, yeast and ferments, thickener (guar gum).
**GF7**	Water, corn starch, rice starch, calcium caseinate (milk proteins), corn flour, rice flour, **extra virgin olive oil**, yeast, apple fiber, salt, thickeners (hydroxypropylmethylcellulose, sodium carboxymethylcellulose, xanthan gum), soy protein, brown cane sugar, preservatives (E-200, E-281), flavour.
**GF8**	Water, corn starch, whole rice flour, **olive oil**, chickpea flour, thickeners (hydroxypropylmethylcellulose, xanthan gum), yeast, sugar, sea salt, antioxidant (extract rich in tocopherols), preservatives (E-282, E-202), emulsifier (E-471), flavour, acidity regulator (citric acid).
**GF9**	Water, corn starch, tapioca starch, rice flour, potato starch, **vegetable oils [sunflower, extra virgin olive (2%)]**, sugar, yeast, thickener (hydroxypropylmethylcellulose), salt, egg white powder, emulsifiers (E-471, E-481), preservative (E-281), raising agents (disodium diphosphate, sodium hydrogen carbonate), ascorbic acid.
**GF10**	Corn starch, water, sourdough (14%, rice flour and water), rice starch, rice syrup, fibers (psyllium, apple), **sunflower oil**, millet flour (2,6 %), soy protein, quinoa flour (1.7%), thickener (hydroxypropylmethylcellulose), yeast , salt, honey.
**GF11**	Water, sourdough (rice flour, water), corn starch, rice flour (5.5%), rice syrup, **sunflower oil**, buckwheat flour (2.7%), thickener (hydroxypropylmethylcellulose), millet flour (1.9%), pea protein, fibers (psyllium, citrus), sea salt, yeast, acidity regulator (tartaric acid), skimmed milk powder without lactose, emulsifiers (E-471, sorbitan esters).
**GF12**	Water, corn starch, rice flour, pea protein, **sunflower oil**, rice syrup, tapioca starch, potato starch, sugar, yeast, corn fiber, inulin, thickener (hydroxypropylmethylcellulose), salt, emulsifiers (E-471, E-472e), flavour, preservatives (E-200, E-282).
**GF13**	Corn starch, water, **egg**, corn flour, sugar, **sunflower oil**, yeast, salt, thickeners (xanthan gum, guar gum), preservatives (E-200).

**GF14**	Water, corn starch, **sunflower oil**, yeast, whole rice flour, dextrose, egg albumin, thickeners (xanthan gum, cellulose), acidity regulator (monocalcium phosphate), raising agent (sodium hydrogen carbonate), salt, emulsifier (E-471), preservatives (E-282, E-202), anti-caking agent (calcium carbonate), flavours and ascorbic acid.
**GF15**	Water, potato starch, corn starch, **sunflower oil**, tapioca starch, egg white powder, rice bran, sugar, yeast, millet flour, thickeners (cellulose, carboxymethylcellulose, hydroxypropylmethylcellulose, xanthan gum), wine vinegar, salt, humectant (glycerol) preservatives (E-200, E-202, E-282), flavour.
**GF16**	Water, gluten-free blend [corn starch, **whole egg powder**, dextrose, thickener (xanthan gum), ascorbic acid, acidity regulator (monocalcium phosphate), salt], yeast, **sunflower oil**, sugar, salt, preservatives (E-282, E-202), apple cider vinegar.
**GF17**	Corn starch, water, **sunflower oil**, yeast, whole rice flour, dextrose, egg albumin, thickeners (xanthan gum, hydroxypropylmethylcellulose, sodium carboxymethylcellulose) acidity regulator (monocalcium phosphate), raising agent (sodium hydrogen carbonate); salt, emulsifier (E-471), preservatives (E-282, E-202), ascorbic acid, flavour.
**GF18**	Gluten-free flour blend [corn starch (55%), corn flour (24%), dextrose, thickeners (xanthan gum, hydroxypropylcellulose, carboxymethylcellulose, guar gum), salt, emulsifiers (E-471, E-472e), bamboo fiber, raising agents (sodium hydrogen carbonate, sodium aluminum phosphates, calcium phosphates), preservative (E-200), ascorbic acid], **sunflower oil**, yeast, humectant (glycerol), water.
**GF19**	Corn starch, **sunflower oil**, sugar, salt, thickeners (xanthan gum, carboxymethylcellulose), bamboo fiber, emulsifiers (E-472e, E-471), raising agents (sodium hydrogen carbonate, sodium aluminium phosphates, calcium phosphates), ascorbic acid, preservative (E-200), water, yeast.
**GF20**	Corn starch, **sunflower oil**, thickeners (xanthan gum; carboxymethylcellulose; hydroxypropylmethylcellulose; guar gum), dextrose, yeast, emulsifiers (E-471, E-472e), salt, humectant (glycerol), bamboo fiber, raising agents (sodium hydrogen carbonate, disodium diphosphate, acid sodium aluminium phosphate, calcium phosphates), preservative (E-200), ascorbic acid, water.

In bold letters the fats added to each bread sample and the ingredients that are an important source of cholesterol (in this case whole egg ingredients, samples GF 1, 2, 4, 13, 16)

Table S5 Nutritional information declared on the labels of gluten-containing (G) sliced white sandwich bread samples, comparison with the analytical results (100 g of edible portion)

**Label information**	**G1**	**G2**	**G3**	**G4**	**G5**	**G6**	**G7**	**G8**	**G9**	**G10**	**G11**	**G12**	**G13**	**G14**
Energy (kcal)	269	274	251	255	251	241	259	259	246	251	251	245	254	254
Fat (g)	4.5	4.5	3	1.9	3	2	3	3	2	3	3	2.2	2.6	2.6
Saturated fat (g)	0.8	0.9	0.5	0.5	0.5	0.5	0.5	0.5	0.4	0.5	0.5	0.4	0.8	0.8
Carbohydrates (g)	46	47	46	49	46	44.9	48	48	47	46	46	46	46	46
Sugars (g)	2.5	3.4	3	5.1	4	2.6	4	5	5	3	4	2.7	3.8	3.8
Dietary fiber (g)	4.7	--	2	2.9	2	3.7	2	--	3	2	--	1.7	3.3	3.3
Protein (g)	8.8	9.6	9	9.4	9	8.9	9	9	9	9	9	9.4	10	10
Salt (g)	1.3	1	1.3	1.3	1.3	0.86	1.5	0.8	1.1	1.3	1.3	0.9	0.97	1.1
**Analytical results and deviation relative to declared contents**														
Fat (g)	3.14	3.01	1.72	1.54	1.97	2.07	1.76	1.68	1.52	1.63	1.61	1.70	2.30	1.57
Deviation in %	-30.2	-33.1	-42.6	-19.0	-34.2	3.3	-41.3	-44.0	-24.1	-45.5	-46.2	-22.5	-11.5	-39.5
Deviation in g	-1.36	-1.49	-1.28	-0.36	-1.03	0.07	-1.24	-1.32	-0.48	-1.37	-1.39	-0.50	-0.30	-1.03
Salt (g)	1.308	1.044	1.393	1.005	1.249	0.895	1.457	1.042	1.002	1.441	1.029	0.992	1.097	1.059
Deviation in %	0.6	4.4	7.1	-22.7	-4.0	4.1	-2.9	30.3	-8.9	10.9	-20.8	10.2	13.1	-3.7
Deviation in g	0.008	0.044	0.093	-0.295	-0.051	0.035	-0.043	0.242	-0.098	0.141	-0.271	0.092	0.127	-0.041
**Percentage contribution to reference nutrient intake (from analytical results)**														
Fat (%)	4.5	4.3	2.5	2.2	2.8	3.0	2.5	2.4	2.2	2.3	2.3	2.4	3.3	2.2
Salt (%)	21.8	17.4	23.2	16.7	20.8	14.9	24.3	17.4	16.7	24.0	17.2	16.5	18.3	17.7

--, not declared. In red deviations that exceed the recommended tolerances for nutrient values declared on the label [5], if fat declared is <10g/100g, maximum tolerance ±1.5g, if fat declared 10-40g, maximum tolerance ±20%, if salt declared <1.25g/100g, maximum tolerance ±0.375g, if salt declared >1.25g/100g, maximum tolerance ±20%. Reference nutrient intakes for fat 70 g and for salt 6g [6].

Table S6 Nutritional information declared on the labels of gluten-free (GF) sliced white sandwich bread samples, comparison with the analytical results (100 g of edible portion)

**Label information**	**GF1**	**GF2**	**GF3**	**GF4**	**GF5**	**GF6**	**GF7**	**GF8**	**GF9**	**GF10**	**GF11**	**GF12**	**GF13**	**GF14**	**GF15**	**GF16**	**GF17**	**GF18**	**GF19**	**GF20**
Energy (kcal)	284	283	252	258	238	262	246	263.13	274	239	244	299	273.82	241	247	260	241	373	246	390.75
Fat (g)	5.8	5.9	4	3.4	2.94	4.9	3.8	6.04	8.4	3.4	4.7	5.3	4.62	5.8	11	3.9	5.8	10.3	5.9	11.18
Saturated fat (g)	3	3.2	1.3	0.59	0.86	2.6	0.7	0.89	1.4	0.5	0.5	0.6	1.02	0.8	1.1	0.54	0.8	1.8	1.2	2.94
Carbohydrates (g)	53	53	48	55.4	50.77	50.2	44.2	48.05	46	45	45	60	55.86	41.3	28	54.5	41.3	64.5	46.8	68
Sugars (g)	7.8	7.6	3.3	0.19	0.58	3.3	0.49	1.47	7.7	3.3	2.2	3.4	2.9	3.8	5	2	3.8	6.7	1	3.28
Dietary fiber (g)	5.8	5.4	0.8	0.25	--	4.1	--	3.16	3.7	7.3	4.8	--	--	4.5	11.5	--	4.5	7.4	--	5.5
Protein (g)	1.7	1.7	6.1	1.5	2.13	2.2	7.2	2.57	1.9	3.5	3.1	3.1	2.2	3.7	5	1.6	3.7	1.9	1.3	1.99
Salt (g)	1.4	1.5	2.9	1.83	0.684	1.2	1.8	1.47	1.37	1	1.2	1.4	1.48	1.5	1.5	1.36	1.47	1.8	1.14	1.75
**Analytical results and deviation relative to declared contents**																				
Fat (g)	4.36	4.77	1.73	1.76	1.99	1.46	2.59	4.91	5.14	1.84	2.69	4.06	2.59	3.95	7.78	3.04	3.93	4.52	4.73	4.26
Deviation in %	-24.8	-19.1	-56.7	-48.4	-32.1	-70.2	-31.9	-18.7	-38.8	-45.9	-42.7	-23.5	-44.0	-31.9	-29.3	-22.1	-32.2	NC	-19.8	NC
Deviation in g	-1.44	-1.13	-2.27	-1.64	-0.95	-3.44	-1.21	-1.13	-3.26	-1.56	-2.01	-1.24	-2.03	-1.85	-3.22	-0.86	-1.87	NC	-1.17	NC
Salt (g)	1.767	1.711	1.371	1.831	0.720	1.578	1.899	1.607	1.357	0.910	1.195	1.257	1.229	1.169	1.220	1.081	1.195	1.805	1.412	1.297
Deviation in %	26.2	14.1	-52.7	0.1	5.3	31.5	5.5	9.3	-0.9	-9.0	-0.4	-10.2	-16.9	-22.1	-18.6	-20.6	-18.7	0.3	23.8	-25.9
Deviation in g	0.367	0.211	-1.529	0.001	0.036	0.378	0.099	0.137	-0.013	-0.090	-0.005	-0.143	-0.251	-0.331	-0.280	-0.279	-0.275	0.005	0.272	-0.453
**Percentage contribution to reference nutrient intake (from analytical results)**																				
Fat (%)	6.2	6.8	2.5	2.5	2.8	2.1	3.7	7.0	7.3	2.6	3.8	5.8	3.7	5.6	11.1	4.3	5.6	6.5	6.8	6.1
Salt (%)	29.4	28.5	22.9	30.5	12.0	26.3	31.6	26.8	22.6	15.2	19.9	20.9	20.5	19.5	20.3	18.0	19.9	30.1	23.5	21.6

--, not declared. NC, not calculated because energy and macronutrients declared on the label are not reliable (in yellow), since the moisture (%) estimated from the nutrients declared greatly differs from moisture analytically determined (GF18, estimated, 14.1, determined, 36.8; GF20, estimated, 11.6, determined 44.0). For all samples the moisture was estimated by the following formula %moisture = 100 -%fat - %carbohydrates - %dietary fiber - %protein - %salt, and compared with the real moisture determined. In red deviations that exceed the recommended tolerances for nutrient values declared on the label [5], if fat declared is <10g/100g, maximum tolerance ±1.5g, if fat declared 10-40g, maximum tolerance ±20%, if salt declared <1.25g/100g, maximum tolerance ±0.375g, if salt declared >1.25g/100g, maximum tolerance ±20% . Reference nutrient intakes for fat 70 g and for salt 6g [6].

References

1. AOAC International (2016) Official methods of analysis of AOAC International, 20th edn. AOAC International, Rockville

2. Guardiola F, Codony R, Rafecas M, Boatella J, López A (1994) Fatty acid composition and nutritional value of fresh eggs, from large- and small-scale farms. J Food Comp Anal 7: 171-188. http://doi.org/10.1006/jfca.1994.1017

3. Royal Decree 1137/1984, of March 28, which approves the Technical sanitary regulation for the manufacture, circulation and trade of bread and special breads, BOE No 146 of June 19, 1984 and its subsequent amendments (consolidated version July 2017)

4. Royal Decree 308/2019, of April 26, which approves the Quality norm of bread, BOE No 113 of May 11, 2019 (into force July 1, 2019)

5. Guidance document for competent authorities for the control of compliance with EU legislation on the provision of food information to consumers. December 2012. https://ec.europa.eu/food/sites/food/files/safety/docs/labelling_nutrition-vitamins_minerals-guidance_tolerances_1212_en.pdf . Accessed 12 April 2020

6. Regulation (EU) 1169/2011 of the European Parliament and of the Council of 25 October 2011 on the provision of food information to consumers. OJEU L304:18-63

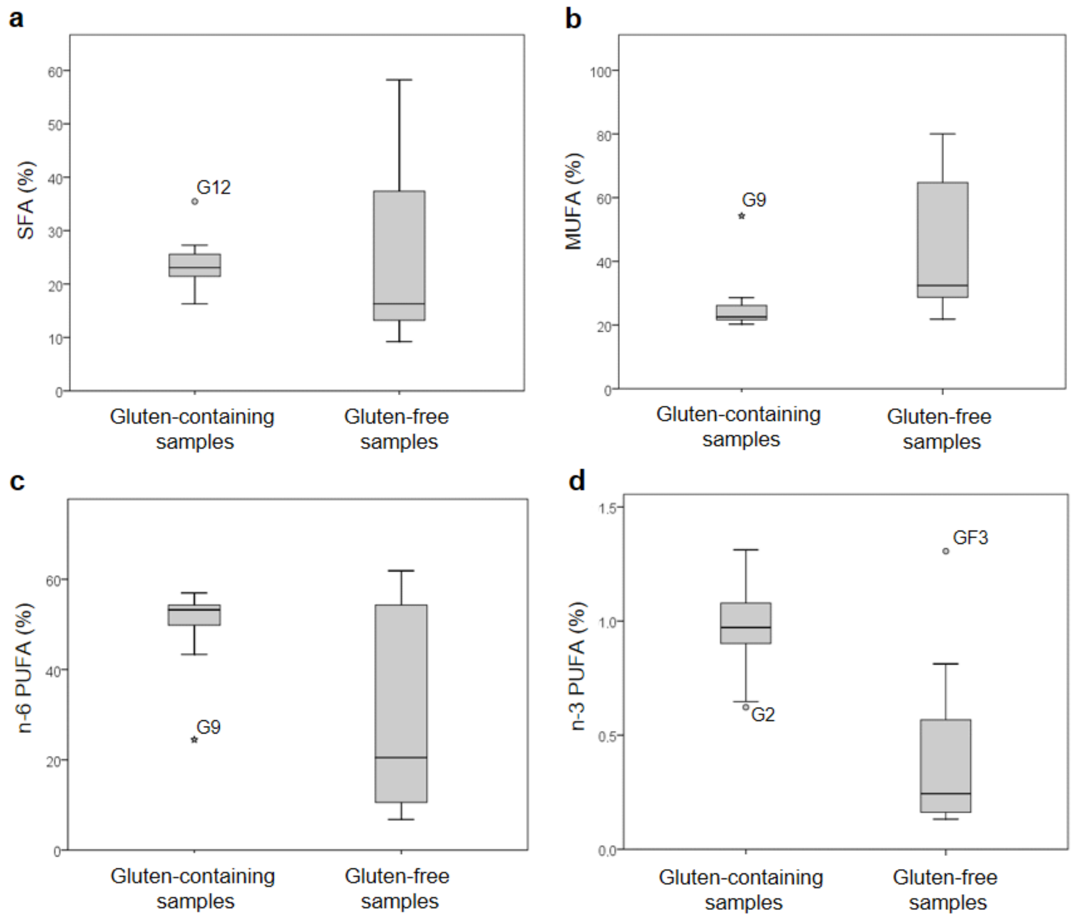

Fig. S1 Distribution of gluten-containing (n = 14) and gluten-free (n = 20) white sandwich bread according to its composition in fatty acids: (**a**) saturated fatty acids, SFA; (**b**) monounsaturated fatty acids, MUFA; (**c**) n-6 polyunsaturated fatty acids, n-6 PUFA; (**d**) n-3 polyunsaturated fatty acids, n-3 PUFA

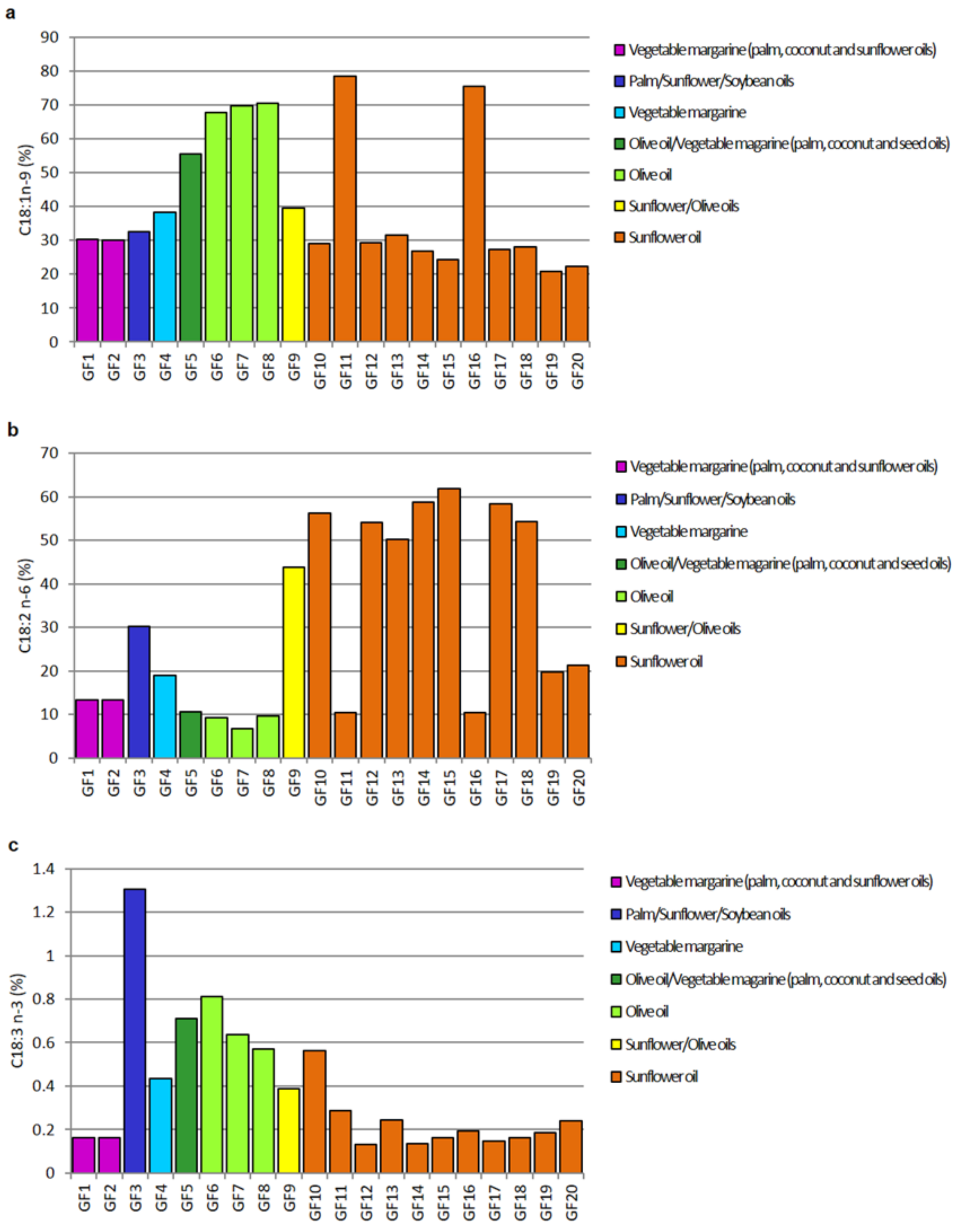

Fig. S2 Contents (%) of (**a**) oleic (C18:1 n-9), (**b**) linoleic (C18: 2 n-6), and (**c**) linolenic (C18: 3 n-3) acids in gluten-free samples (n = 20), colored according to the type of fat declared in the list of ingredients

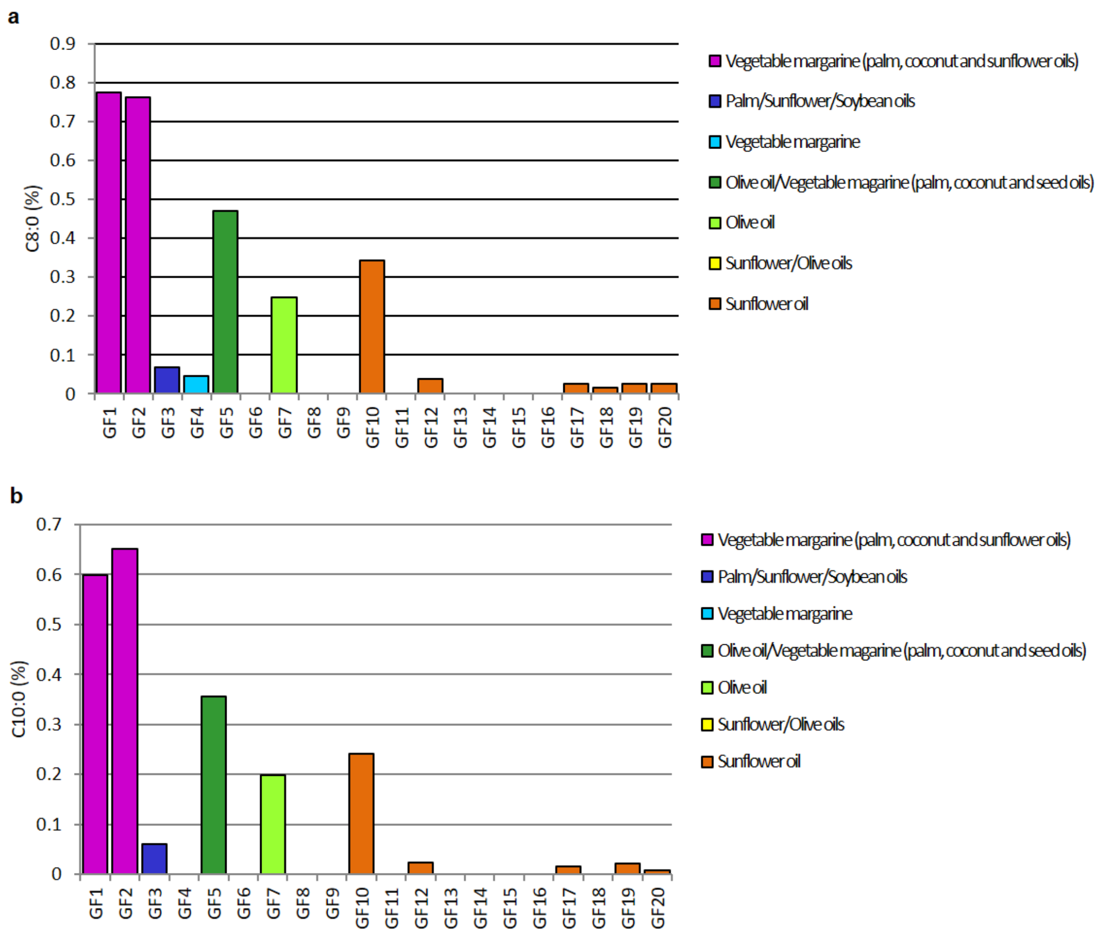

Fig. S3 Contents (%) of (**a**) caprylic (C8:0) and (**b**) capric (C10:0) acids in gluten-free samples (n = 20), colored according to the fat declared in the list of ingredients

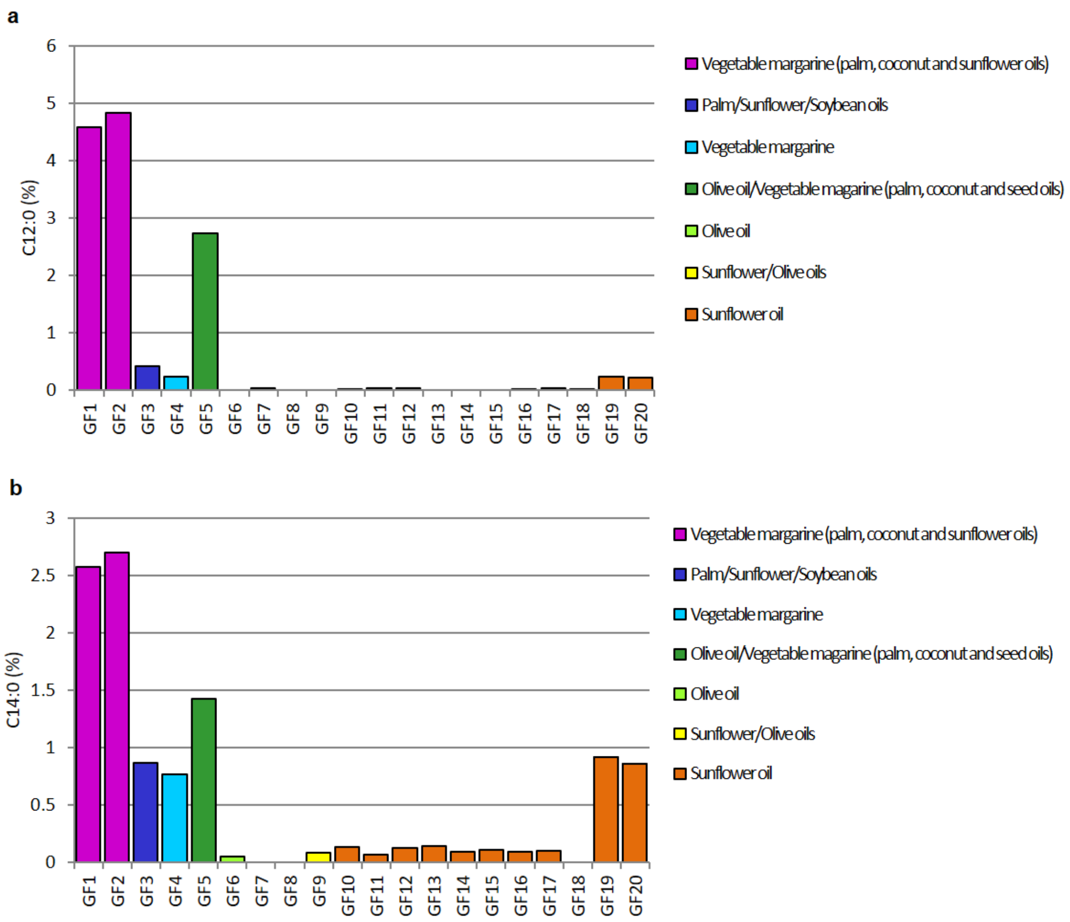

Fig. S4 Contents (%) of (**a**) lauric (C12:0) and (**b**) myristic (C14: 0) acids in gluten-free samples (n = 20), colored according to the fat declared in the list of ingredients

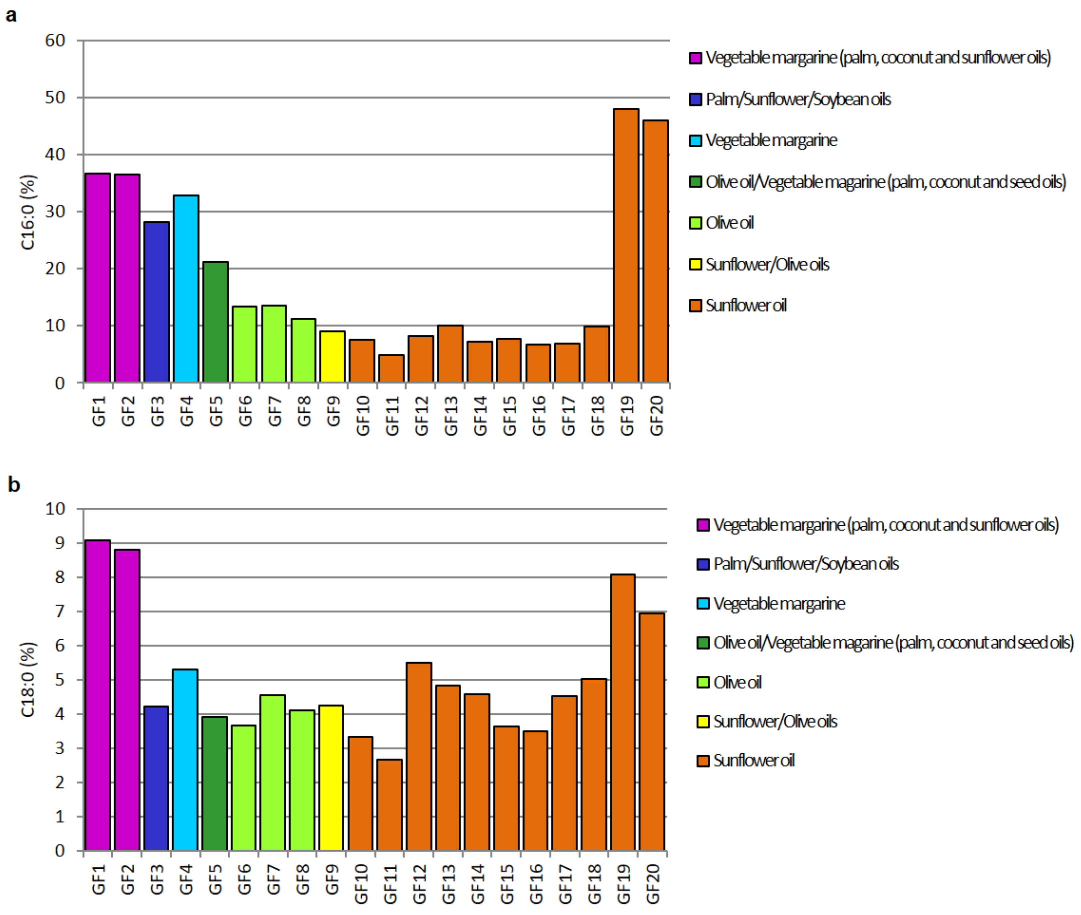

Fig. S5 Contents (%) of (**a**) palmitic (C16: 0) and (**b**) stearic (C18: 0) acids in gluten-free samples, colored according to the fat declared in the list of ingredients

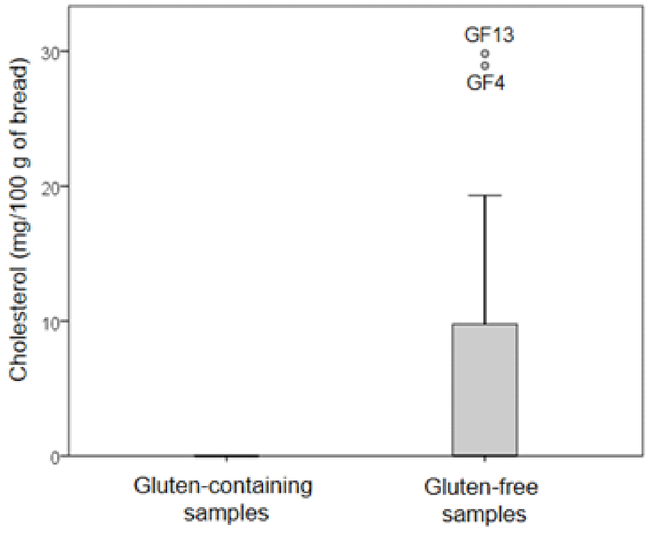

Fig. S6 Distribution of gluten-containing (n = 14) and gluten-free (n = 20) white sandwich bread according to its cholesterol content

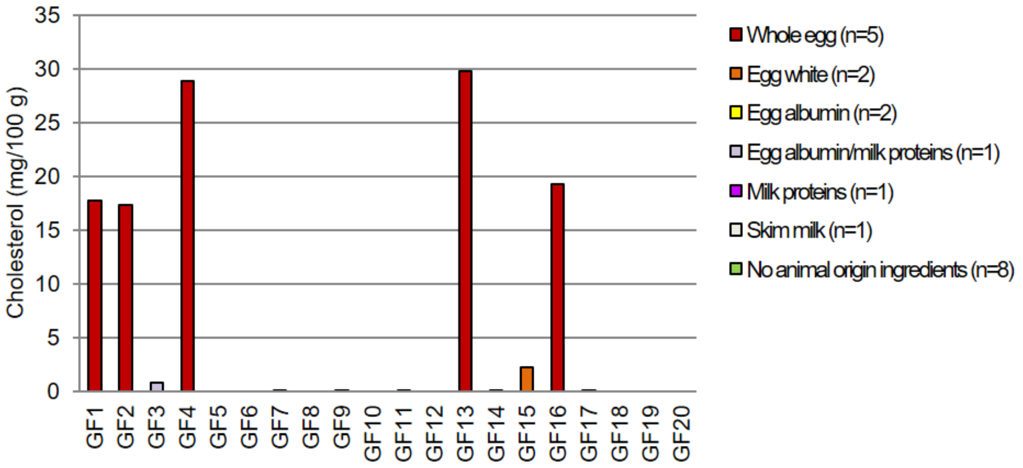

Fig. S7 Cholesterol content (mg/100 g) in gluten-free samples, colored according to the possible sources of cholesterol declared in the list of ingredients

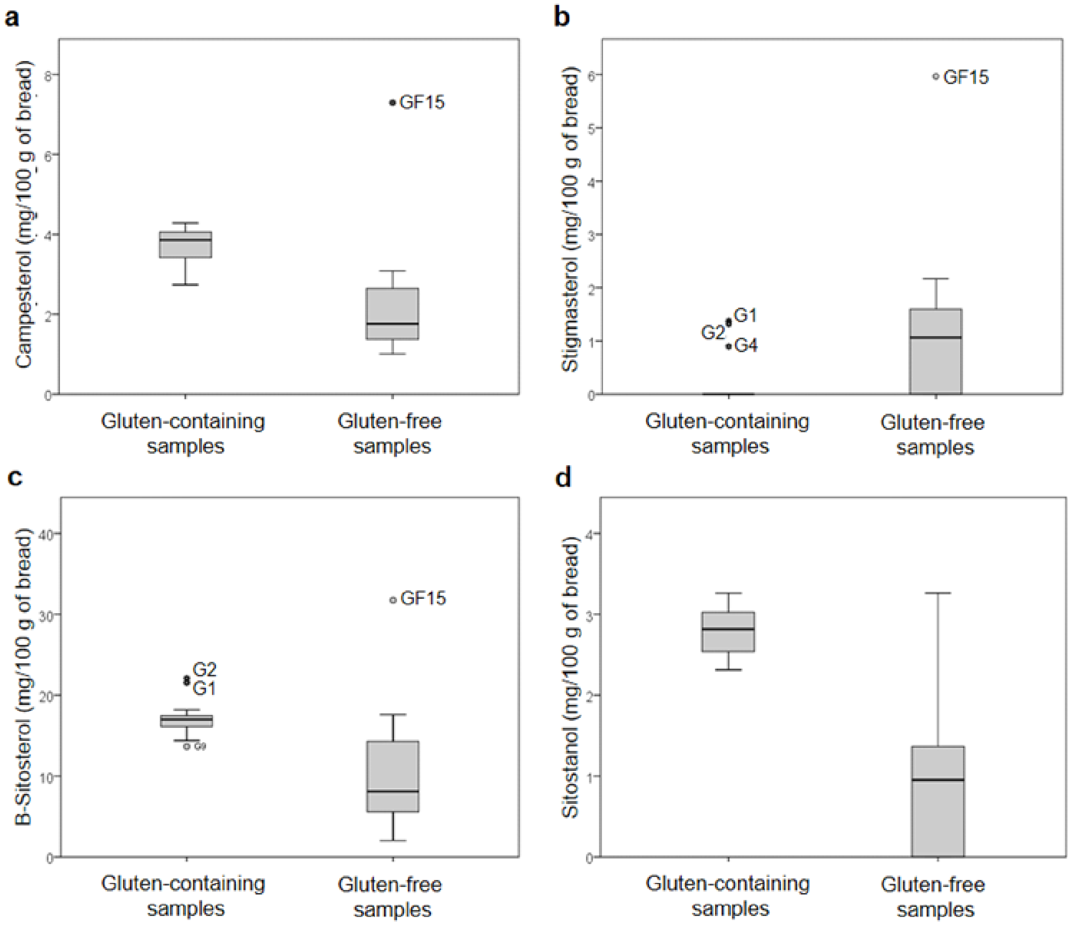

Fig. S8 Distribution of gluten-containing (n = 14) and gluten-free (n = 20) white sandwich bread according to its composition in phytosterols: (**a**) campesterol; (**b**) stigmasterol; (**c**) β-sitosterol; (**d**) sitostanol

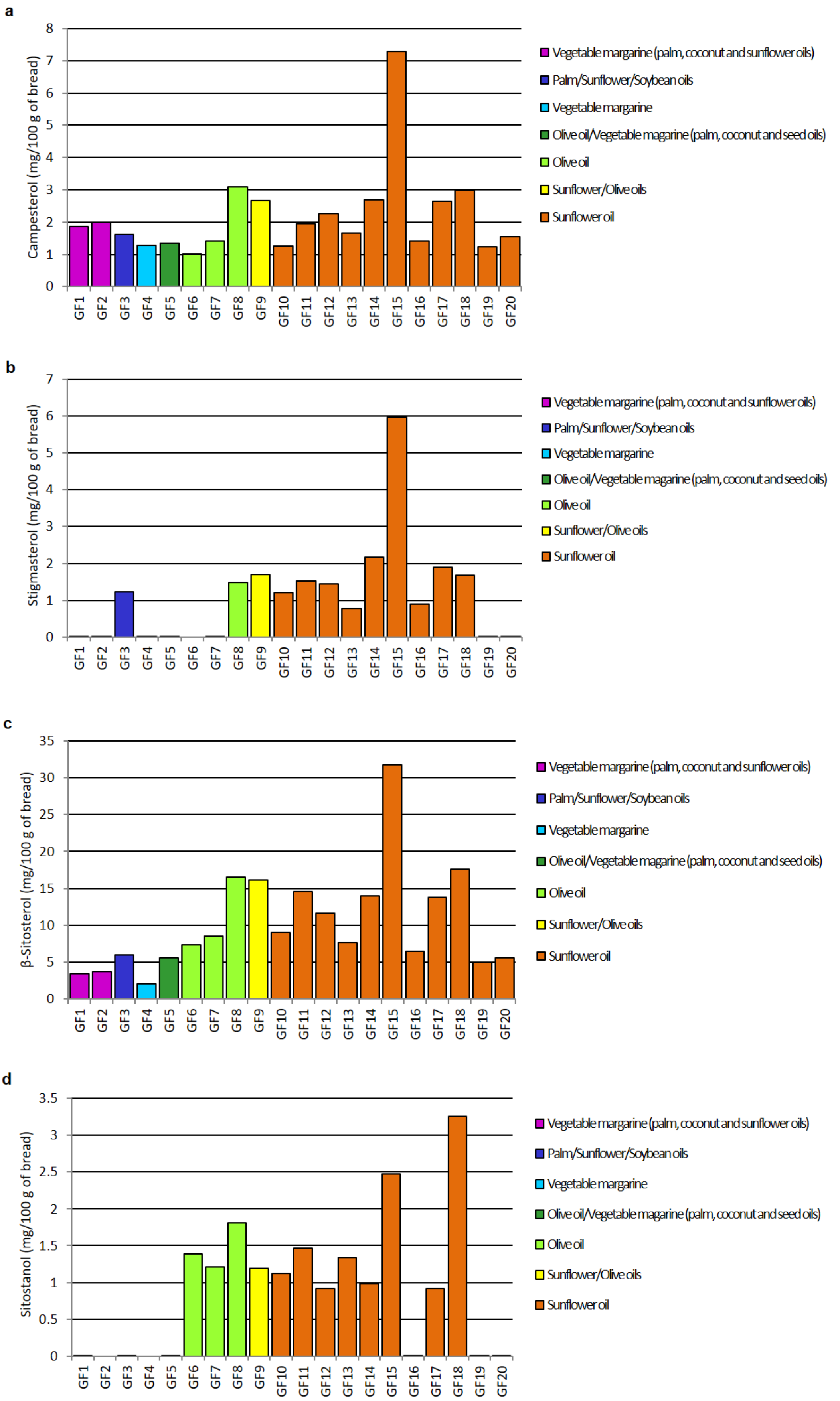

Fig. S9 Contents (mg/100 g) of (**a**) campesterol, (**b**) stigmasterol, (**c**) β-sitosterol and (**d**) sitostanol in gluten-free samples, colored according to the fat declared in the list of ingredients

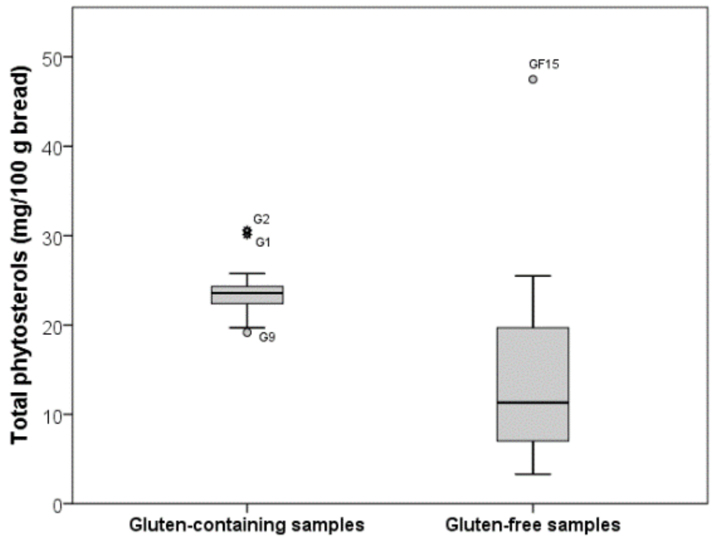

Fig. S10 Distribution of gluten-containing (n = 14) and gluten-free (n = 20) white sandwich bread according to its total phytosterol content
